# Caspase-1 and the inflammasome promote polycystic kidney disease progression

**DOI:** 10.3389/fmolb.2022.971219

**Published:** 2022-11-29

**Authors:** Katherine I. Swenson-Fields, Christopher J. Ward, Micaila E. Lopez, Shaneann Fross, Anna L. Heimes Dillon, James D. Meisenheimer, Adib J. Rabbani, Emily Wedlock, Malay K. Basu, Kyle P. Jansson, Peter S. Rowe, Jason R. Stubbs, Darren P. Wallace, Michael P. Vitek, Timothy A. Fields

**Affiliations:** ^1^ The Jared J. Grantham Kidney Institute, University of Kansas Medical Center, Kansas City, KS, United States; ^2^ Department of Anatomy and Cell Biology, University of Kansas Medical Center, Kansas City, KS, United States; ^3^ Department of Internal Medicine, Division of Nephrology and Hypertension, University of Kansas Medical Center, Kansas City, KS, United States; ^4^ Department of Pathology and Laboratory Medicine, University of Kansas Medical Center, Kansas City, KS, United States; ^5^ Duke University Medical Center, Durham, NC, United States; ^6^ Resilio Therapeutics LLC, Durham, NC, United States

**Keywords:** Caspase-1, hydroxychloroquine, IL-1β, IL-18, inflammasome, MYC, polycystic kidney disease, YAP

## Abstract

We and others have previously shown that the presence of renal innate immune cells can promote polycystic kidney disease (PKD) progression. In this study, we examined the influence of the inflammasome, a key part of the innate immune system, on PKD. The inflammasome is a system of molecular sensors, receptors, and scaffolds that responds to stimuli like cellular damage or microbes by activating Caspase-1, and generating critical mediators of the inflammatory milieu, including IL-1β and IL-18. We provide evidence that the inflammasome is primed in PKD, as multiple inflammasome sensors were upregulated in cystic kidneys from human ADPKD patients, as well as in kidneys from both orthologous (*PKD1* ^*RC/RC*^ or RC/RC) and non-orthologous (*jck*) mouse models of PKD. Further, we demonstrate that the inflammasome is activated in female RC/RC mice kidneys, and this activation occurs in renal leukocytes, primarily in CD11c+ cells. Knock-out of *Casp1*, the gene encoding Caspase-1, in the RC/RC mice significantly restrained cystic disease progression in female mice, implying sex-specific differences in the renal immune environment. RNAseq analysis implicated the promotion of MYC/YAP pathways as a mechanism underlying the pro-cystic effects of the Caspase-1/inflammasome in females. Finally, treatment of RC/RC mice with hydroxychloroquine, a widely used immunomodulatory drug that has been shown to inhibit the inflammasome, protected renal function specifically in females and restrained cyst enlargement in both male and female RC/RC mice. Collectively, these results provide evidence for the first time that the activated Caspase-1/inflammasome promotes cyst expansion and disease progression in PKD, particularly in females. Moreover, the data suggest that this innate immune pathway may be a relevant target for therapy in PKD.

## Introduction

Autosomal dominant polycystic kidney disease (ADPKD) is the most common monogenic kidney disease, with an estimated prevalence of around 1 in 1000 individuals (Lanktree et al., 2018), and is the fourth leading cause of renal failure (Spithoven et al., 2014). ADPKD is caused primarily by inherited mutations in one of two genes, *PKD1* (∼78% of cases) and *PKD2* (∼15%) (Cornec-Le Gall et al., 2018). The disease progresses slowly and variably, resulting in renal failure for roughly half of patients with ADPKD by the sixth decade (Cornec-Le Gall et al., 2018). The disease is accompanied by acute and chronic pain for most (60%) patients (Gabow, 1993; Bajwa et al., 2004). Currently, there is only one FDA-approved treatment for ADPKD, the vasopressin V2-receptor antagonist, Jynarque (tolvaptan) (Torres, 2018). However, because of adverse side effects of tolvaptan, there remains a need to develop safe, effective new therapies for PKD.

ADPKD is characterized by numerous renal cysts derived from the nephron that expand continuously over the patient’s lifetime, causing massive enlargement of the kidney. These expanding cysts, many of which originate in the collecting duct, compress and distort the surrounding parenchyma, including the microvasculature, causing chronic ischemic and obstructive renal injury. Massive fibrosis develops in these kidneys, further contributing to the compressive injury, which ultimately results in compromised renal function (Grantham, 2003; Grantham et al., 2011). Cystic changes are also accompanied by ongoing inflammation and the presence of innate and adaptive immune cells in large numbers, some of which have been shown to modulate disease progression (for review, see (Zimmerman et al., 2020). Using rodent models of PKD, we and others have used experimental methods to deplete renal mononuclear phagocytes, including infiltrating and resident macrophages and dendritic cells, to demonstrate that these cells contribute to cyst cell proliferation and cyst expansion (Karihaloo et al., 2011; Swenson-Fields et al., 2013; Yang et al., 2018).

To discover new treatment strategies for ADPKD, we sought to understand the pathophysiologic mechanisms that promote disease progression, reasoning that this approach could identify common key molecular processes that could be therapeutic targets. A number of seemingly disparate stimuli have been shown to accelerate cystic disease in PKD model rodents. These include exposure to commensal microbes or microbial products (Werder et al., 1984; Gardner et al., 1986; Gardner et al., 1987), renal ischemia-reperfusion (IR) injury (Patel et al., 2008; Takakura et al., 2009; Kurbegovic and Trudel, 2016), and the deposition of renal calcium oxalate (CaOx) or calcium phosphate (CaP) crystals (Torres et al., 2019). One feature, that is, common to these stimuli is that each has been shown to activate the Caspase-1 inflammasome (Werder et al., 1984; Gardner et al., 1986; Gardner et al., 1987; Melnikov et al., 2001; Shigeoka et al., 2010; Mulay et al., 2013). Potential effects of Caspase-1/inflammasome activation on PKD progression have not previously considered.

The Caspase-1 inflammasome is a multi-protein scaffold, the sole known purpose of which is activation of Caspase-1. Once activated, Caspase-1 cleaves multiple cellular substrates, including gasdermin D, pro-IL-1β, and pro-IL-18. Cleaved gasdermin D promotes the formation of plasma membrane pores, which then allows the release of cleaved, active IL-1β and IL-18, and may also trigger pyroptosis (Carty et al., 2019). There are far-reaching inflammatory sequelae of IL-1β and IL-18 release. IL-1β in particular is a master regulatory cytokine that influences many cell types to promote the transcription of hundreds of genes, including those encoding both inflammatory cytokines and chemokines (Weber et al., 2010; Anders, 2016). This amplifies inflammation further by promoting the infiltration and activation of neutrophils, dendritic cells, monocytes, and lymphocytes.

Inflammasome activation has been well characterized mainly in innate immune cells. Components of the inflammasome have been found to be expressed in other renal cells, including endothelial cells, mesangial cells, podocytes, and tubular epithelial cells, but convincing evidence demonstrating the hallmarks of canonical inflammasome activation in these cells when isolated from primary sources (i.e., cleavage of Caspase-1 and extracellular release of IL-1β) is lacking or inconsistent (Anders, 2016). The inflammasome assembles as an innate immune response triggered by both microbial products (microbe-associate molecular patterns, MAMPs), produced by both commensal and pathogenic microbes, and also host-derived cellular products released in response to stress, tissue injury, or cell death (damage-associated molecular patterns, DAMPs). MAMPs include bacterial products such as toxins, peptidoglycans, flagellin, outer membrane components (e.g., LPS) and nucleic acids, including those from RNA and DNA viruses (Anders and Muruve, 2011). DAMPs include products present in all intracellular compartments (e.g., ATP, heat shock proteins, histones, HMGB1, mtDNA), as well as extracellular matrix breakdown products (e.g., biglycan, hyaluronan, fibrinogen) and crystals of all types (e.g., urate, cholesterol, CaOx, CaP, and adenine) (Roh and Sohn, 2018). Stimuli known to promote PKD progression, i.e., exposure to commensal microorganisms, renal IR, and the induction of CaOx and CaP renal crystals, all generate MAMPs and DAMPs that have been shown to promote Caspase-1 inflammasome formation and activation and play a role in mediating the resulting inflammatory renal injury and/or systemic effects (Li et al., 1995; Melnikov et al., 2001; Wang et al., 2005; Mulay et al., 2014).

MAMPs and DAMPs engage germ-line encoded pattern recognition receptors (PRRs) present both on the plasma membrane and in the cytosol to initiate formation of the inflammasome complex, typically by promoting self-oligomerization through homotypic molecular interactions. These oligomers then recruit the adaptor protein, ASC (apoptosis-associated speck-like protein containing a caspase recruitment domain), and pro-Caspase-1, which then self-cleaves to become active. Some PRRs can be activated by both MAMPs and DAMPs, thereby promoting signaling pathways common to both types of stimuli. These PRRs include the toll-like receptors (TLRs) present on membranes, as well as the cytosolic inflammasome sensors, which include the NLRP (nucleotide-binding oligomerization domain, leucine-rich repeat and pyrin, domain containing) group of proteins, the most well-known of which is NLRP3 (Anders et al., 2004; Anders and Muruve, 2011). Other sensors known to activate Caspase-1 in both humans and mice also include other NLRP-related proteins (Nlrp1, 2, 3, 4, 6,12, and Nlrc4), IPAF, a sensor for bacterial flagellin, AIM2, a sensor for dsDNA, and MEFV (aka Pyrin), a specific MAMP sensor (Broz and Dixit, 2016; Christgen et al., 2020). NLRP3 is best known among the inflammasome sensors because it is activated in response to myriad MAMPs and DAMPs and contributes to a wide variety of inflammatory diseases (Mangan et al., 2018).

Most DAMPs and MAMPs, including those that engage the inflammasome sensors, act first by interacting with TLRs, especially TLR2, 4, and 6 (Roh and Sohn, 2018, which are among the many TLRs expressed by both immune cells and non-immune cells, including renal tubular epithelial cells (Anders, 2004 #6). Binding of TLRs results in the activation of NF-kB-mediated transcription, which acts directly or indirectly to drive the upregulation of both inflammasome sensors and inflammasome components (e.g., *NLRP1*, *NLRP3*, and *Casp1*) to facilitate formation and activation of the inflammasome complex in a process known as “priming” (Lee et al., 2015; Christgen et al., 2020). TLR-activated NF-kB can also directly drive expression of some pro-inflammatory cytokines and chemokines (Liu et al., 2017).

As renal cysts expand and promote injury in PKD, DAMP generation likely occurs. A primary DAMP for both human and mouse PKD is likely to be extracellular ATP (eATP). eATP is released by cells in response to a wide range of stimuli including mechanical stress, cell membrane damage, inflammation and hypoxia (Anders and Muruve, 2011). Notably, renal epithelial cells from patients with ADPKD or from PKD mouse models have been shown to release 5 times higher levels (high-nanomolar to micromolar quantities) of extracellular ATP than cells from healthy controls (Wilson et al., 1999; Schwiebert et al., 2002). In addition, high levels of ATP have been identified in the cyst fluids of PKD kidneys from both human and rodents (Wilson et al., 1999; Palygin et al., 2018). eATP is a well-known stimulator of the NLRP3 inflammasome and acts by binding the ionotropic P2X7 receptor, which is highly expressed in immune cells and is also present in epithelial cells (Di Virgilio et al., 2018; Kopp et al., 2019). Signaling by eATP *via* this receptor stimulates K+ efflux, which is a required step for NLRP3 inflammasome activation (Munoz-Planillo et al., 2013). Whether these elevated levels of ATP promote activation of the NLRP3 inflammasome in PKD has not been studied.

Our hypothesis is that, in addition to mediating effects of experimental stimuli that promote cystic disease, the Caspase-1 inflammasome is likely to be operative during the natural course of PKD, promoting its progression. In these studies, we test this hypothesis using both animal models and analysis of human ADPKD tissue. We demonstrate for the first time that the canonical Caspase-1/inflammasome is primed in both human ADPKD and in both orthologous and non-orthologous mouse models of disease and is activated primarily in immune cells. Further, we provide evidence that activated Caspase-1 promotes PKD progression in female mice and that it can be effectively targeted to ameliorate disease.

## Results

### Inflammasome components and products are elevated in kidneys from autosomal dominant polycystic kidney disease patients

**FIGURE 1:**
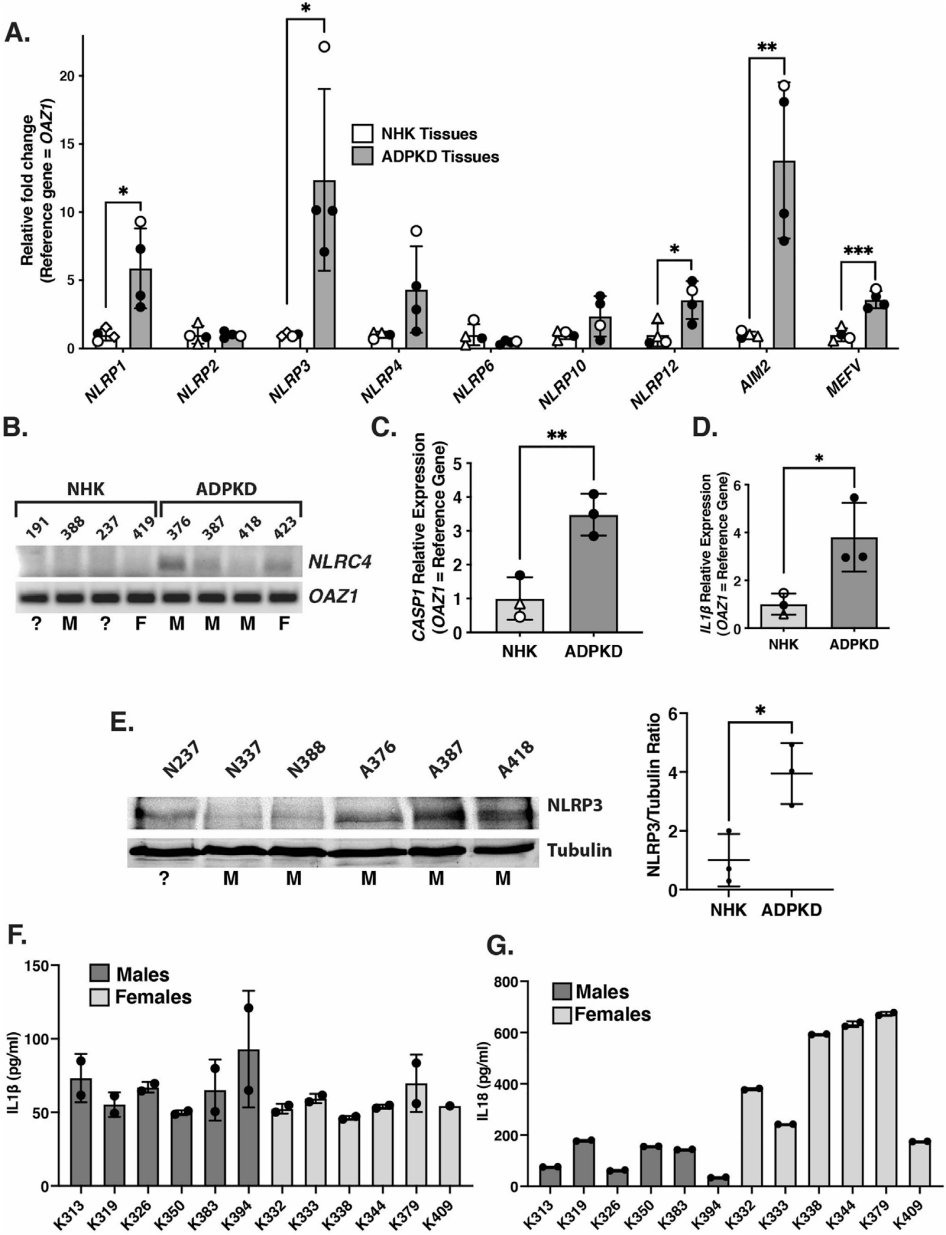
Expression of inflammasome sensors and components are elevated in kidneys from ADPKD patients. **(A)** qRT-PCR of transcripts encoding inflammasome sensors from kidneys of patients with ADPKD or from non-cystic human kidneys (NHK). Open circles = females; closed circles = males; open triangles = unknown sex. **(B)** Semi-quantitative PCR products of *NLRC4* and reference gene (*OAZ1*) from cDNAs of transcripts from the same kidney samples as in **(A)**. Assigned kidney numbers are shown for each NHK and ADPKD sample. M = male; F = female; ? = unknown sex. **(C,D)** qRT-PCR of transcripts *CASP1* **(C)** and *IL1β* **(D)** from ADPKD and NHK kidney tissues samples. Symbols indicating sex are the same as in **(A)**. **(E)** Western blot of NLRP3 and tubulin from NHK and ADPKD kidney protein samples. Assigned kidney numbers and sex are shown. On right is graph showing the relative NLRP3/Tubulin protein levels measured from the blot. **(F,G)** Concentrations of IL-1β **(F)** and IL-18 **(G)** in duplicate samples of cyst fluids collected from individual male and female patients with ADPKD measured by ELISA.

To determine whether inflammasome priming occurs in PKD, the relative transcript levels of ten inflammasome sensors known to promote Caspase-1/inflammasome activation, as well as *CASP1* and *IL1B,* were assessed first in the kidneys of ADPKD patients vs. non-cystic human kidneys (NHK) (Figures 1A–D). Transcripts encoding multiple sensors, including NLRP1, NLRP3, NLRP12, AIM2, MEFV, NLRC4, and *CASP1* and *IL1B* were significantly elevated in kidneys from ADPKD patients relative to NHK. In addition, Western blot of the NLRP3 sensor showed increased levels of this protein in these cystic tissues (Figure 1E). We were unable to unambiguously detect Caspase-1 protein (uncleaved or cleaved) on western blots of whole kidney extracts, however (data not shown), which may be due to technical issues with the antibodies or may reflect the expression of this protease in a restricted subset of renal cells [http://humphreyslab.com/SingleCell/ (Wu et al., 2019; Kirita et al., 2020)].

To further evaluate inflammasome activation in the kidneys of ADPKD patients, we measured the levels of IL-1β and IL-18, which are primary products of activated Caspase-1, in the cyst fluids collected from 12 different patients (Figures 1F,G). This analysis revealed measurable levels of both cytokines (ranging from 46 to 93 pg/ml for IL-1β and 34–673 pg/ml for 1L-18) in all samples. These findings are similar to those obtained in previous studies (Gardner et al., 1991; Parikh et al., 2012). While there are non-canonical Caspase-1-independent mechanisms of generating the extracellular presence of IL-1β and IL-18, these mechanisms operate primarily under inflammatory conditions in which neutrophils are the primary infiltrate (Afonina et al., 2015; Netea et al., 2015), which is not the case in cystic kidneys of ADPKD patients (TAF, unpublished). Thus, in spite of our inability to detect cleaved Caspase-1 protein in whole kidney extracts, our collective results showing both inflammasome priming and the presence of IL-1β and IL-18 suggest that inflammasome activation is likely occurring in the kidneys of ADPKD patients.

We evaluated the potential contribution of cystic epithelial cells from the kidneys of ADPKD patients to inflammasome activation. In previous studies, ADPKD cyst nephrons were shown to have elevated transcript expression of a number of inflammasome components, including IL-1β and IL-18 (de Almeida et al., 2016). In addition, renal tubular epithelial cells have been shown to express both NLRP3 and Caspase-1 transcripts and proteins, although whether these cells are capable of inflammasome activation and release of IL-1β is questionable (for reviews (Anders, 2016; Kim et al., 2019). Using human ADPKD cyst cells and tubular epithelial cells isolated from NHK grown in culture, we found a number of transcripts encoding sensors and inflammasome components that were elevated in cyst cells, suggesting that priming of the inflammasome had occurred (Supplementary Figure S1A–D). Notably the pattern of elevated transcripts only partially overlapped that found in whole human kidney tissues. NLRP3 protein was detected in these cells but, unlike renal tissues, there was no difference in the levels present in ADPKD *vs.* NHK cells (Supplementary Figure S1E). In addition, little to no IL-1β was detected in the conditioned media from either cell type (Supplementary Figure S1F). These results indicate that while ADPKD cyst cells in culture are primed (or partially primed) inflammasome activation is not ongoing.

We tested whether ADPKD cyst cells could activate the NLRP3 inflammasome using established methods to maximally prime and trigger this activation (Guzova et al., 2019). ADPKD cyst cells were treated with agonists Pam3CSK4 and LPS specific for the membrane receptors, TLR2 and TLR4, respectively, which are known to be present on these cells and to interact with same MAMPs and many DAMPs present *in vivo* to bring about priming (Roh and Sohn, 2018; Christgen et al., 2020). These cells were then treated with a potassium ionophore, nigericin, known to rapidly and potently force activation of the NLRP3 inflammasome in primed cells, causing Caspase-1 cleavage and release of IL-1β (Guzova et al., 2019). While treatment of ADPKD cyst cells with these MAMPs was sufficient to promote further upregulation of *CASP1* and *IL1B* transcripts, there was no change in the level of Caspase-1 protein, and the addition of potassium ionophore did not result in cleavage of Caspase-1 or the release of IL-1β (Supplementary Figure S1G–J). In contrast, similar treatment of a control monocytic cell line (THP-1 cells) performed in parallel resulted in Caspase-1 cleavage and IL-β release, as expected (Supplementary Figure S1I,K). These results suggest that while inflammasome priming mechanisms can occur in ADPKD cyst cells, their ability to activate the NLRP3 inflammasome appears unlikely. Thus, inflammasome activation in human ADPKD kidneys is likely occurring in cells other than the cyst epithelial cells. We examined this hypothesis further using an orthologous mouse model of PKD.

### Inflammasome components are elevated in kidneys from orthologous and non-orthologous mouse models of polycystic kidney disease

**FIGURE 2:**
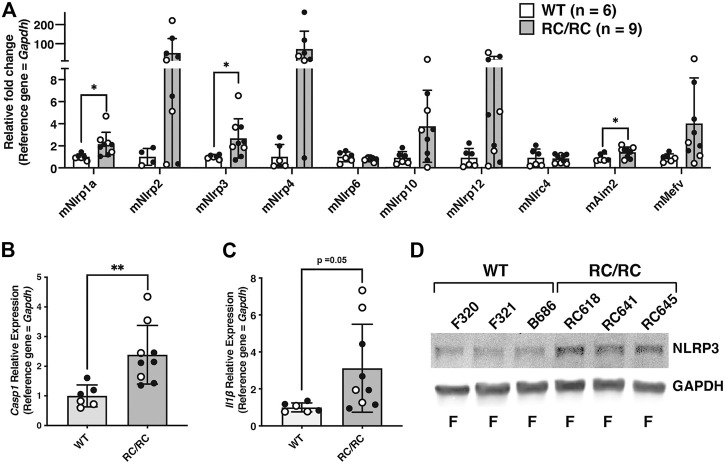
Expression of inflammasome sensors and components are elevated in kidneys from RC/RC mice. **(A)** qRT-PCR of transcripts encoding inflammasome sensors from kidneys of RC/RC or WT mice at 6 months of age. Open circles = females; closed circles = males. qRT-PCR of transcripts *Casp1* **(B)** and *Il1b* **(C)** from RC/RC and WT kidney tissues samples. Symbols indicating sex are the same as in **(A)**. **(D)** Western blot of NLRP3 and GAPDH from WT and RC/RC kidney protein samples. Assigned kidney numbers and sex are shown.

To begin analysis of inflammasome activation in an animal model of PKD, the relative expression levels of inflammasome sensors and components were assessed in the kidneys of an orthologous mouse model of ADPKD, *Pkd1* ^*RC/RC*^ (or “RC/RC” mice). These mice have a knock-in *Pkd1* missense allele, *Pkd1* (p.R3277C) or “RC,” that matches an allele found in a human family with cystic disease (Hopp et al., 2012). On the C57BL/6 background these RC/RC mice develop detectable renal cysts by 3 months of age, which slowly and progressively enlarge with time. At 6 months, RC/RC mice exhibit many well-developed renal cysts, although no loss of kidney function has yet occurred (Arroyo et al., 2021). As in the kidneys of human ADPKD patients, there was significant upregulation of transcripts for multiple sensors in the RC mice kidneys relative to WT, including those encoding NLRP1a, NLRP3, and AIM2 (Figure 2A). These kidneys also showed elevated expression of *Casp1* transcripts and trended toward an upregulation of *Il1b* as well (Figures 2B,C). There was also increased expression of the NLRP3 sensor protein in RC/RC *vs.* WT kidneys, similar to that seen in renal tissue of human ADPKD patients (Figure 2D). As for human tissue, we were unable to detect Caspase-1 protein in whole kidney extracts by western blotting (data not shown). Regardless, these results suggest that inflammasome priming is ongoing in RC/RC mice at this stage.

To evaluate whether the mutations in *Pkd1* (RC/RC mice) or *PKD1* and *PKD2* (humans) were specifically responsible for the elevated expression of inflammasome components, the relative transcript levels encoding sensors, Caspase-1, and IL-1β were assessed in the kidneys of mice with juvenile cystic kidney (*jck*) disease and the non-cystic heterozygotes from the same breeding group at PN38. PKD in *jck* mice arises due to a homozygous mutation in the gene *Nek8*, which encodes a cilia-associated kinase (Liu et al., 2002). While there is formation and growth of renal cysts in the early life of *jck* mice, they do not begin to lose kidney function until after ∼ PN38 (Smith et al., 2006). Results from qRT-PCR showed significant upregulation of transcripts encoding the sensors NLRP3, NLRP10, and MEFV, as well as those encoding Caspase-1 and IL-1β, in the kidneys of *jck* mice relative to the noncystic controls (Supplementary Figure S2A–C). These results indicate that inflammasome priming, and potentially activation, is also ongoing in the kidneys of *jck* mice, similar to that seen for RC/RC mice and patients with ADPKD. These results also suggest that *PKD1* and *PKD2* mutations are not directly responsible for inflammasome priming and activation.

### CD11c+ macrophages and dendritic cells are responsible for inflammasome activation in RC/RC cystic kidneys

Inflammasome activation has been reported to occur in a number of different cell types, although it is primarily associated as an activity of immune cells (Anders, 2016). In cystic PKD kidneys, inflammasome activity has not been previously assessed. However, single cell RNA sequencing in mouse kidneys injured by other means (ischemia-reperfusion, unilateral ureter obstruction, and allograft rejection) has demonstrated that Caspase-1 is most upregulated in monocytes, macrophages, and dendritic cells [http://humphreyslab.com/SingleCell/(Wu et al., 2018; Wu et al., 2019; Kirita et al., 2020)]. Given this observation and our inability to detect Caspase-1 cleavage in whole RC/RC kidney extracts, we examined whether inflammasome activation was occurring in the renal immune cells of RC/RC mice.

**FIGURE 3:**
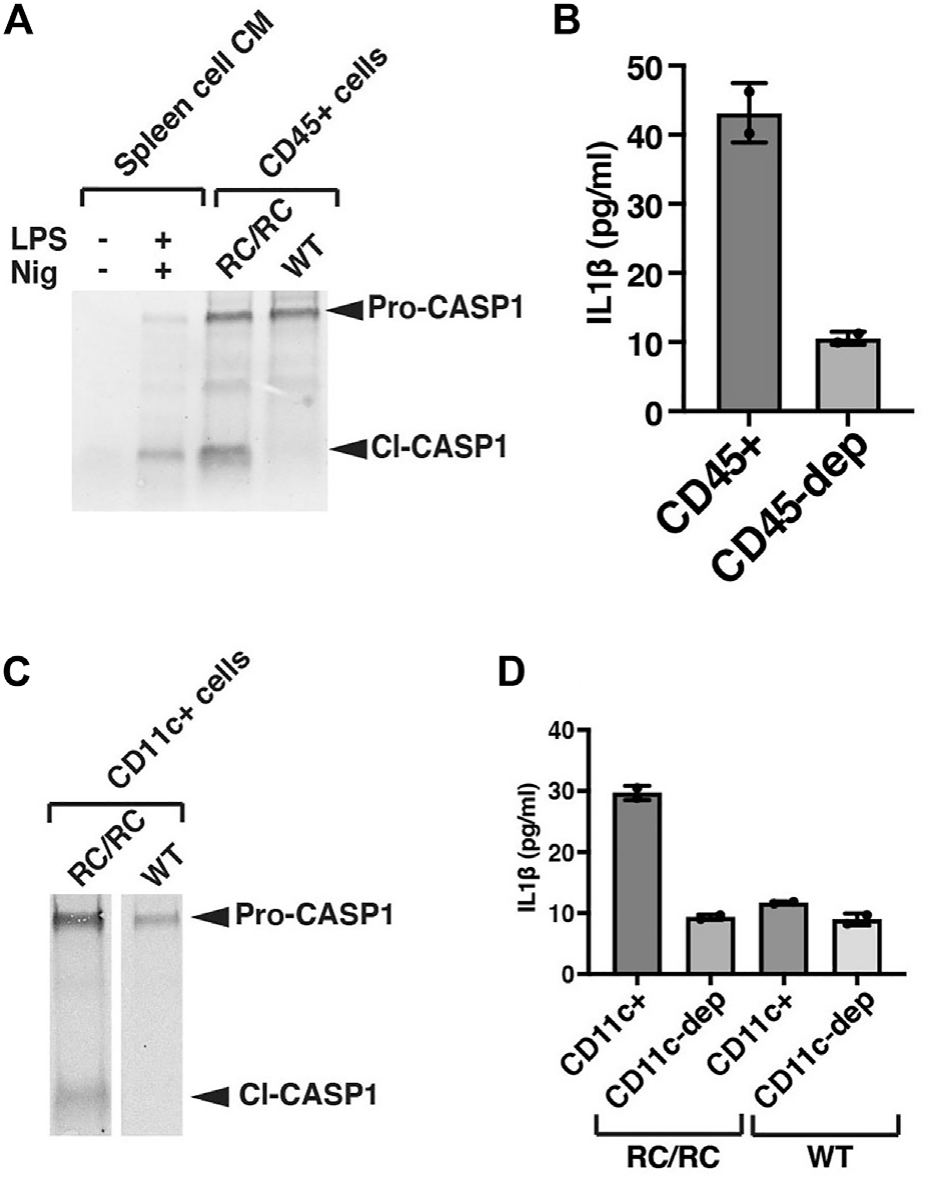
CD11c+ macrophages and dendritic cells are responsible for inflammasome activation in RC/RC cystic kidneys. **(A)** CD45^+^ MACS (magnetic cell separation) cells were purified from single kidneys of female RC/RC or WT mice prior to immunoblot analysis (100,000 cells/lane) using an antibody specific for Caspase-1. This experiment was carried out on 4 separate occasions with similar results. As control samples, single cells were prepared from mouse spleens and either treated or not *in vitro* with LPS and nigericin to stimulate inflammasome activation. Conditioned media from these cells were collected and concentrated prior to immunoblot analysis. **(B)** CD45^+^ cells were purified from single kidneys of female RC/RC mice by MACS column as in **(A)** and equal numbers (100,000) of these cells and those left after passing through the column (CD45-depleted, dep) were seeded in 96-well plates and incubated overnight prior to the collection of conditioned media and the measurement of IL-1β by ELISA. This experiment was carried out on 3 separate occasions with similar results. **(C)** CD11c+ MACs cells were purified from single kidneys of female RC/RC and 2 kidneys/experiment from female WT mice and subjected to immunoblot analysis (100,000 cells/lane) as in **(A)**. **(D)** CD11c+ cells purified by MACS column as in **(C)**, and equal numbers (100,000) of these cells and those left after passing through the column (CD11c-depleted, dep) were seeded in 96-well plates and incubated overnight prior to the collection of conditioned media and the measurement of IL-1β by ELISA. This experiment was carried out on 3 separate occasions with similar results.

Initially, we assessed inflammasome activity in total renal leukocyte populations. For these experiments, we prepared single-cell suspensions from individual kidneys of female WT or cystic RC/RC mice and used Ficoll gradients to enrich for leukocytes. Using this method, we obtained an average of ∼ 2–3 × 10^6^ leukocyte-enriched cells for each single RC/RC kidney for further purification, whereas only ∼1/3 of this number was typically obtained from WT kidneys (data not shown). Flow cytometry analyses revealed ∼70%–90% CD45^+^ cells in these preparations from both RC/RC and WT (data not shown). CD45^+^ is a tyrosine phosphatase expressed on the plasma membrane of all mature hematopoietic cells, except erythrocytes and platelets (Nakano et al., 1990). CD45^+^ cells include macrophages, dendritic cells, T and B lymphocytes, and innate lymphoid cells. The CD45^+^ cells were isolated from the Ficoll-enriched preparations using anti-CD45 antibody-coupled magnetic beads and were assessed for the presence of Caspase-1 by western blot. Uncleaved pro-Caspase-1 was readily detectable in equal numbers of CD45^+^ cells from both RC/RC and WT kidneys. However, only those cells isolated from RC/RC kidneys showed the presence of the cleaved, activated form of this enzyme (Figure 3A). These results suggest that the inflammasome is activated in the CD45^+^ leukocytes of RC/RC but not WT kidneys.

To determine whether these RC/RC renal leukocytes are producing IL-1β, CD45^+^ cells were isolated from cystic kidneys and cultured overnight, and IL-1β was measured in the conditioned media (CM) by ELISA (Figure 3B). In parallel, equal numbers of leukocyte-enriched cells following removal of the CD45^+^ population (CD45-depleted) were also cultured. Post-isolation flow analyses in these experiments demonstrated high efficiency of the depletion: >90% of the cells isolated with the anti-CD45 magnetic beads were positive for CD45, but only 1%–4% of the CD45-depleted cells were positive (data not shown). As show in Figures 3A,B relatively high-level of IL-1β (43 pg/ml in this experiment) was measured in the CM of CD45^+^ isolated cells compared to that from the CD45-depleted cells (11 pg/ml), which was below the lowest IL-1β standard (15.6 pg/ml) in this assay.

In efforts to more narrowly identify those leukocytes in which the inflammasome is activated in RC kidneys, we focused on CD11c + cells. CD11c is a marker both of dendritic cells and macrophage populations in the kidney (Weisheit et al., 2015; Viehmann et al., 2018), and these populations have been found to be primarily responsible for renal inflammasome activation in the setting of kidney injury types other than PKD (Mulay et al., 2013). Treatment of mice with clodronate lipsosomes, such as that used previously to restrain cystic disease progression in mouse models of PKD, has been shown to deplete renal CD11c + cells (Mulay et al., 2013), suggesting potential pro-cystic effects of these cells. Flow analysis of the leukocyte-enriched populations from RC/RC and WT kidneys showed around 30%–40% of these cells were CD11c+ (data not shown).

Renal CD11c+ cells were isolated from the Ficoll-enriched cell populations of female RC/RC and WT mice with magnetic anti-CD11c beads and assessed for Caspase-1 cleavage and IL-1β production. There was a reduced efficiency of isolation of CD11c+ cells compared to that obtained with the anti-CD45 antibody reagents: ∼70%–85% of the cells isolated by anti-CD11c beads from both RC/RC and WT kidneys were CD11c +, while ∼5%–15% of the CD11c-depleted cells were CD11c+ (data not shown). As expected, there was a high percentage of CD45^+^ cells present in both the anti-CD11c bead-isolated and CD11c-depleted cells (greater than 95% and 70%–90% respectively, not shown). Western blots of Caspase-1 showed that uncleaved Caspase-1 was present in anti-CD11c-isolated cells from both RC/RC and WT kidneys, but only RC/RC kidneys showed cleaved Caspase-1 in these preparations (see Figure 3C for an example). In addition, while elevated levels of IL-1β were produced by renal anti-CD11c isolated cells from RC mice, very little IL-1β was detected in equal numbers of CD11c+ isolated cells from WT mouse kidneys or by the CD11c-depleted cells from either mouse type (see Figure 3D for an example), despite the high percentage of CD45^+^ cells in this depleted population. These experiments indicate that inflammasome activation in the kidneys of RC/RC mice is occurring predominantly in leukocytes, and that CD11c+ cells are likely the primary immune cells responsible for this activation.

### Caspase-1 deficiency in RC mice restrains cystic disease progression in females but not males

Since multiple types of kidney insults that promote inflammasome activation also are known to promote cystic disease progression in rodent models of PKD, we hypothesized that a genetic deficiency of *Casp1* in RC/RC mice to restrict inflammasome activation might restrain cyst expansion during the natural course of the disease. Using TALENs technology the *Casp1* gene was mutated to create several deletions (Supplementary Figure S3A–C), one of which was bred to homozygosity in the RC/RC mouse. This mutation (Del3, Supplementary Figure S3B) which has a 28 bp deletion that includes the coding region for the active site cysteine residue of Caspase-1 and the splice donor of exon 6, results in a knockout (KO) genotype for expression of the gene (Supplementary Figure S3D).

**FIGURE 4:**
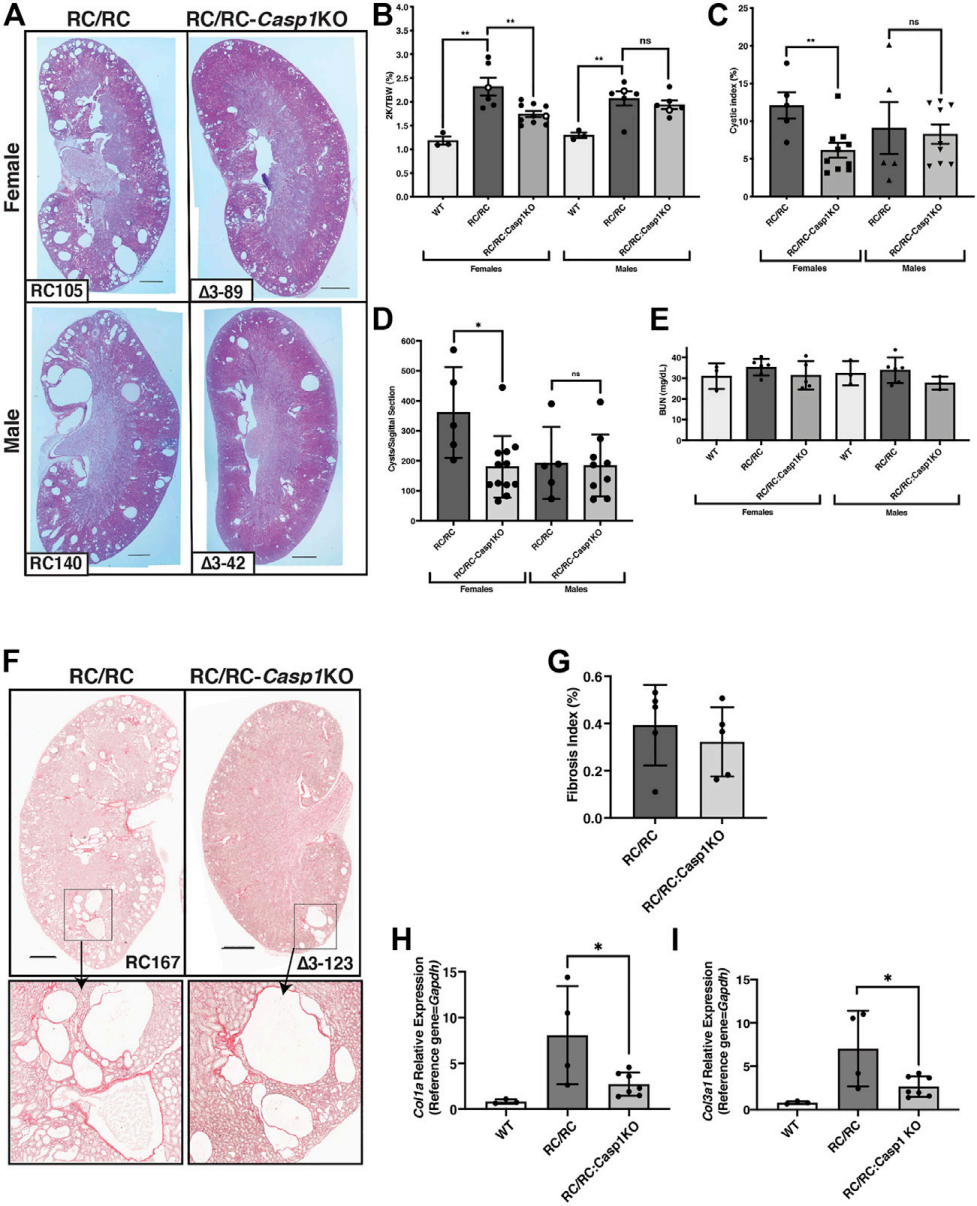
*Casp1*-deficiency restrains cystic disease progression in female RC/RC mice. **(A)** Formalin-fixed average sized kidneys from 6 m old RC/RC and RC/RC:*Casp1*KO mice were sectioned and stained with hematoxylin and eosin. Scale bars = 1 mm. **(B)** The two-kidney/total body weight percentage (2K/TBW %) was determined for each animal analyzed and plotted as a function of genotype for females and males. Open symbols indicate data points from those mice from which the H&E images shown in **(A)** were taken. The cystic index **(C)** and the number of cysts per sagittal section **(D)** for RC/RC and RC/RC:*Casp1*KO mice were determined and plotted as a function of gender and genotype. **(E)** Blood urea nitrogen (BUN) was determined for each mouse tested and plotted as a function of gender and genotype. **(F)** Sections from kidneys of female RC/RC and RC/RC:*Casp1KO* mice were stained with picrosirius red and illuminated by bright field to visualize collagen type I-α1 (*Col1α1*) and collagen type III-α1 (*Col3α1*). Scale bars = 1 mm. Enlarged images of the boxed regions are shown below as indicated by the arrows. **(G)** The fibrosis index was determined from picrosirius red-stained kidney sections from the RC/RC and RC/RC:*Casp-1*KO female mice. Quantitative RT-PCR of RNA isolated from WT, RC/RC, and RC/RC:*Casp-1*KO female mice, showing the relative expression of the fibrosis markers *Col1α1* **(H)** and *Col3α1* **(I)**.

WT mice, RC/RC mice, and RC/RC mice homozygous for *Casp1* KO (RC/RC:*Casp1*KO) were euthanized at 6 months of age and assessed for parameters of cystic disease progression. These included microscopic examination of H&E-stained, formalin-fixed renal mid-sagittal sections and determination of the 2 kidney/total body weight (2K/TBW) ratios, cystic index, cyst number, and renal function, as estimated by serum blood urea nitrogen (BUN). H&E-stained sections of average-sized kidneys for both females and males showed robust cystic disease in RC/RC mice that appeared to be restrained in the RC/RC:*Casp1*KO mice, particularly in females which showed apparent smaller and fewer cysts (Figure 4A). As shown previously for this strain, the 2K/TBW was elevated in both male and female RC mice compared to WT at this age. The RC/RC:*Casp1*KO mice showed reduced 2K/TBW compared to RC/RC mice but only in females (Figure 4B). Similarly, the cystic index and number of cysts/sagittal sections were also reduced in female RC/RC:*Casp1*KO mice but not the males (Figures 4C,D). The BUN of the RC/RC mice at this age was not elevated, as has been shown previously (Arroyo et al., 2021), and these values were unaltered in the RC/RC:*Casp1*KO mice (Figure 4E).

We assessed the *Casp1* KO effects on renal fibrosis initially by collagen (type1-α1 and type III-α1) staining of sections from fixed samples with picrosirius red (Figure 4F) Because of the predominantly female effect of *Casp1* KO on cyst number and kidney size, we concentrated this analysis on female samples. There was little fibrotic area in the RC/RC kidneys at this age as has been shown previously (Arroyo et al., 2021), and quantitation of the fibrosis indices of these samples showed no significant difference between the RC/RC and RC/RC:*Casp*1KO kidneys (Figure 4G). To detect more subtle potential effects of *Casp1* KO on fibrosis pathways in these kidneys, the relative transcript expression of *Col1α1* and *Col3α1* were determined by qRT-PCR (Figures 4H,I). The expression of these fibrosis transcript markers was elevated in the RC/RC kidneys when compared to WT and was significantly reduced by *Casp1* KO. These results suggest that early renal fibrotic pathways in the female RC/RC mice at this age are restrained by *Casp1* KO.

Sections of these female mouse kidneys were also stained for the proliferation marker, Ki67, which showed very few positive cells (Supplementary Figure S4A). Quantification of the Ki67 + cells lining the cysts and those in the interstitium was carried out and showed no significant difference between the RC/RC and RC/RC:*Casp1*KO samples (Supplementary Figure S4B,C). Finding no differences in proliferation, particularly for the cyst-lining cells, despite the differences in cystic index and cyst numbers, is likely due to the overall low numbers of proliferating cells in these kidneys, coupled with the inadequacies of a snap-shot assessment at a single time point.

### Gene expression profiles of RC/RC and RC/RC:*Casp1*KO kidneys

**FIGURE 5:**
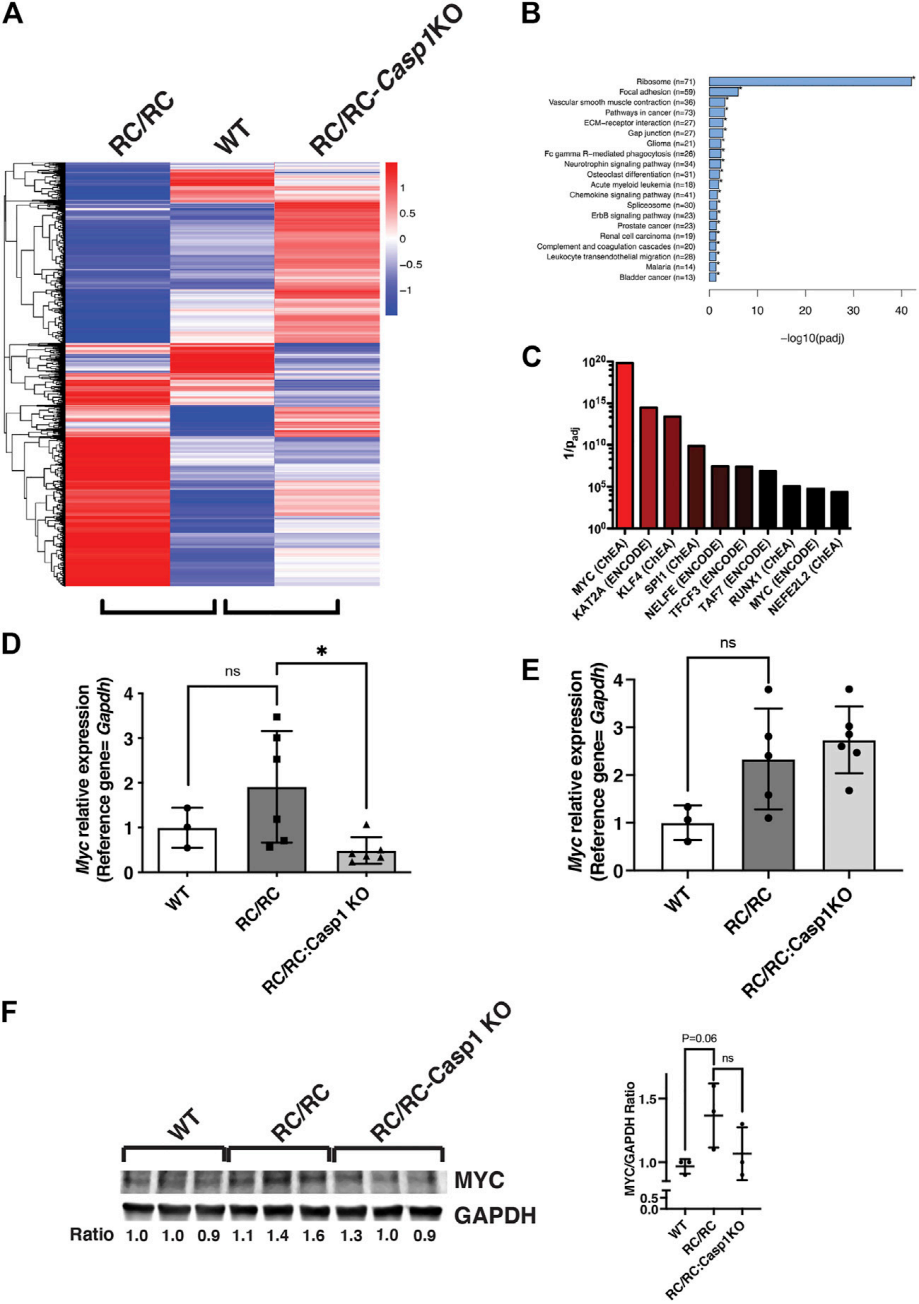
The MYC pathway in RC/RC mice is downregulated by *Casp1* KO in females but not males. **(A)** RNA was isolated from kidneys of 6 month-old RC/RC, WT, and RC/RC:*Casp1*KO mice (2 males and 2 females per group). RNAseq was performed, and hierarchical cluster analysis of differentially expressed genes was used to compare gene expression patterns from each group. Hierarchical clustering analysis was carried out with the log10(FPKM+1) of union differential expression genes of all comparison groups. Differential expression analysis between two conditions/groups was done by using the DESeq2 R package, with the significance criterion being p_adj_ < 0.05. Color descending from red to blue indicates log10(FPKM+1), from large to small. **(B)** ClusterProfiler software was used to perform KEGG (Kyoto Encyclopedia of Genes and Genomes) pathway enrichment analysis from the RNAseq data (males and females). Shown are pathways significantly enriched among all the 3162 DEGs in RC/RC vs. RC/RC:*Casp1KO* kidneys. Pathways are ranked by -log10 (p_adj_). Shown in parentheses are the number of genes mapped to each pathway. **(C)** Genes significantly downregulated in male and female RC/RC:*Casp1KO* kidneys compared to RC/RC kidneys were analyzed using the web-based tool ENRICHR (see text for references). Transcription factors that are present in ENCODE and ChEA databases were matched with consensus target genes from within this gene set. The *y*-axis represents 1/p_adj_. **(D and E)** qRT-PCR of *Myc* transcripts from kidneys of 6 month-old WT, RC/RC, and RC/RC:*Casp1*KO female **(D)** and male **(E)** mouse kidneys (*p < 0.05; *t* test). **(F)** Western blots of MYC and GAPDH from whole protein samples isolated from the kidneys of 6 month-old WT, RC/RC and RC/RC:*Casp1*KO female mice (3 each). The ratio of MYC/GAPDH is shown beneath each sample and is plotted in the graph to the right.

To elucidate the cellular pathways influenced by *Casp1* KO to restrain cyst formation and cyst growth in PKD, gene expression profiling following RNASeq was carried out using both male and female kidneys from WT, RC/RC, and RC/RC:*Casp1*KO mice (see Data Availability Statement). Hierarchical cluster analysis of expressed genes from these kidneys reveals distinct expression profiles (Figure 5A). Notably, many of the gene expression changes seen in RC/RC mice compared to WT are reversed in RC/RC:*Casp1*KO compared to WT. Examination of kidney gene expression in individual mice reveals a similar theme (Supplementary Figure S5), although: 1) female RC/RC mice had a somewhat different pattern of upregulated and downregulated genes, compared to males; and 2) at least a subset of genes downregulated by *Casp1* KO in females appeared to be more variably affected by *Casp1* KO in males. Due to the small number of mice in each group, subsequent RNAseq analyses were performed using the combined male/female data, unless otherwise noted, though key genes were assessed and validated using qRT-PCR in individual males and females separately.

The differentially expressed genes (DEGs) that were upregulated in RC/RC vs. WT kidneys included genes in pathways that have been previously reported in PKD (Chen et al., 2008; Song et al., 2009; Happe et al., 2011; Pandey et al., 2011; Menezes et al., 2012; Dweep et al., 2013; de Almeida et al., 2016; Chatterjee et al., 2017; Malas et al., 2017; Cai et al., 2018; Kunnen et al., 2018; Terabayashi et al., 2020). Upregulated DEGs mapped to Gene Ontology or KEGG (Kyoto Encyclopedia of Genes and Genomes) pathways that are associated with proliferation and growth, such as cAMP, WNT, Hedgehog, Hippo, and TGFβ, as well as pathways involved in epithelial-to-mesenchymal transition and fibroblast proliferation and activation (Supplementary Table S1). Strikingly, there were numerous upregulated genes mapped by Gene Ontology analysis to more than 270 different pathways related to various innate and adaptive immune responses, including immune cell activation, function, and migration and response to cytokines (Supplementary Table S2). Pathways related to pyroptosis and IL-β and IL-18 production, secretion, and response are including among these inflammatory pathways.

Gene expression profiles were compared between RC/RC and RC/RC:*Casp1*KO kidneys to discover those pathways altered by the absence of *Casp1* and particularly those that might influence cystic disease progression. KEGG pathway analysis of all the 3164 DEGs in RC/RC vs. RC/RC:*Casp1KO* kidneys showed that the ribosome pathway was the most altered (Figure 5B and Supplementary Table S3). This pathway included 71 DEGs, mostly encoding ribosome proteins, all of which were downregulated (70 significantly) in the RC/RC:*Casp1*KO kidneys compared to those of RC/RC. A reduction in the expression of these proteins would be expected to diminish ribosome biogenesis and slow growth, which may contribute to the restrained cystic disease in these mice.

### Caspase-1 knockout in female but not male RC mice restrains MYC and YAP pathways, which are central mediators of kidney pathogenesis in polycystic kidney disease.

Since ribosomal genes are downregulated by *Casp1* KO, we focused the next stage of analysis on DEGs that were downregulated in the RC/RC:*Casp1KO* kidneys compared to RC/RC. These DEGs were expected to be associated with cystic disease-promoting pathways that are downregulated by *Casp1* KO. This gene set, which comprises 1405 DEGs, was used to perform ChIP enrichment analysis (ChEA) utilizing a publicly available, online tool, Enrichr (Lachmann et al., 2010; Chen et al., 2013; Kuleshov et al., 2016; Xie et al., 2021), to identify transcription factors that might regulate these genes. In this analysis, MYC was identified as strongly associated with DEGs downregulated in the RC/RC:*Casp1KO* kidneys (Figure 5C and Supplementary Table S4). MYC is a transcription factor, that is, known to promote expression of ribosomal proteins and ribosome biogenesis. Importantly, MYC is also known to be a central node responsible for the promotion of tubular epithelial cell proliferation and cystogenesis in PKD (Kurbegovic and Trudel, 2020). qRT-PCR assessment of renal *Myc* expression in female mouse kidneys showed an uptrend in the levels of this transcript in RC/RC vs. WT mice at this disease stage, which was significantly reversed by *Casp1* KO (Figure 5 D). In male mouse kidneys, while there was also an uptrend in *Myc* expression in RC/RC vs. WT mice similar to that found in females, there was no effect on the levels of this transcript elicited by *Casp1* KO (Figure 5E). The levels of MYC protein in whole kidneys of female mice were assessed by Western blot, which showed increased levels of this protein in RC/RC vs. WT kidneys and reduced levels (2 out of 3 cases) in the RC/RC:*Casp1*KO vs. RC/RC kidneys (Figure 5F). These results suggest that KO of *Casp1* in female RC/RC mice may dampen the MYC pathway in these PKD animals.

**FIGURE 6:**
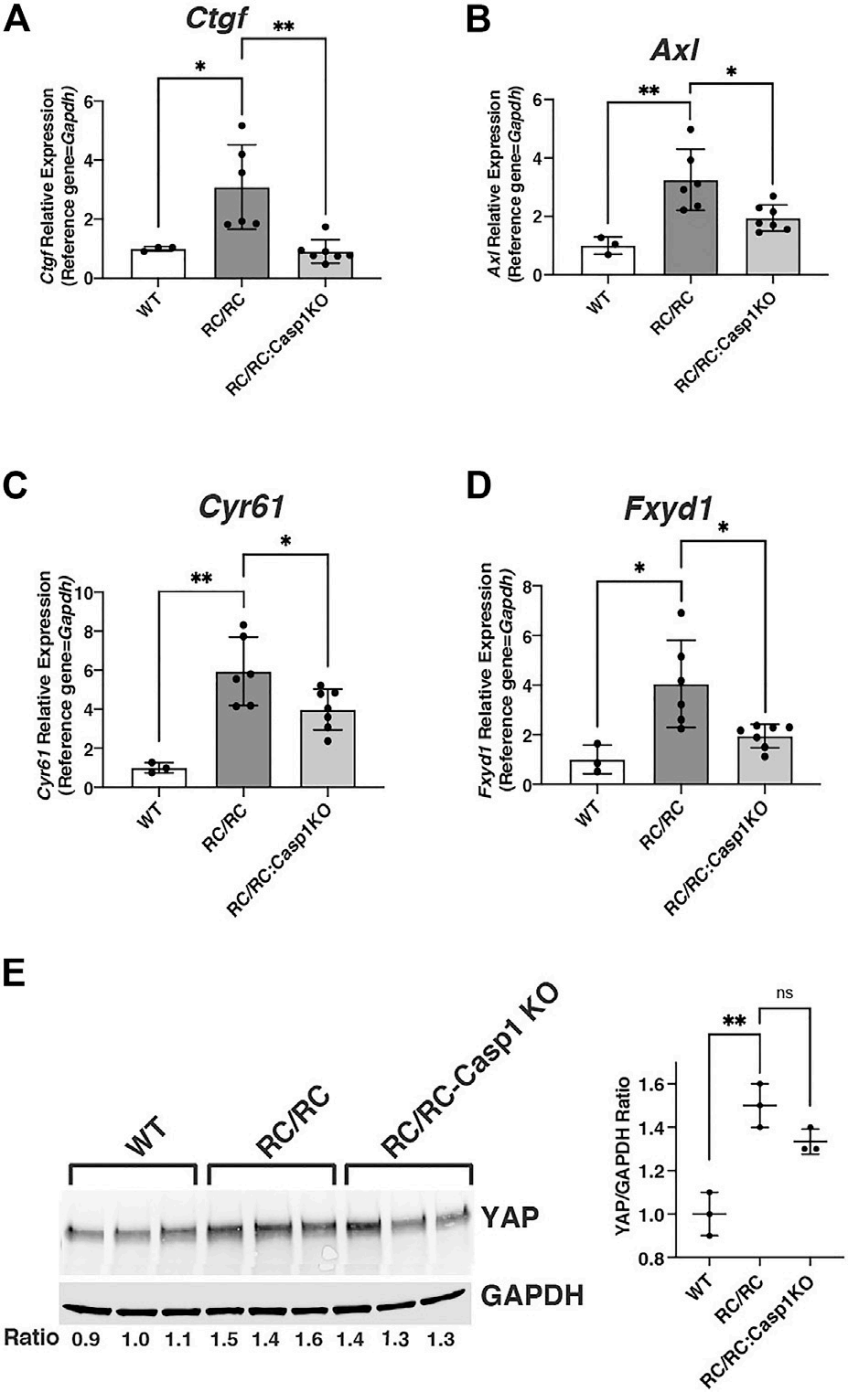
YAP pathway in RC/RC female mouse kidneys is downregulated by *Casp-1* KO. **(A–D)** qRT-PCR of YAP transcript targets from WT, RC/RC and RC/RC:*Casp-1*KO female mouse kidneys: **(A)** *Ctgf*; **(B)** *Axl*; **(C)** *Cyr61* and **(D)** *Fxyd1.* **(E)** Western blots of YAP and GAPDH from protein samples of WT, RC/RC, and RC/RC:*Casp1*KO female kidneys (3 each).

MYC is a transcriptional target of the oncoprotein and transcription coactivator YAP, and the YAP-MYC signaling axis has been found to be a mediator of cystic kidney pathogenesis in a human-orthologous PKD mouse model (Cai et al., 2018). It was of interest then to determine whether the DEGs that were downregulated in the RC/RC:*Casp1*KO vs. RC/RC kidneys were enriched in YAP targets as well as MYC targets. Comparison of these downregulated genes with YAP targets previously identified by ChIP-seq in mouse embryonic stem cells (Lian et al., 2010) revealed that these targets comprised ∼38% of the 1405 DEGS downregulated in the RC/RC:*Casp1*KO kidneys vs. RC/RC (Supplementary Table S5). This list includes key YAP targets upregulated in human ADPKD cystic tissue compared to minimally cystic tissue, such as *Axl*, *Ctgf*, *Cyr61*, and *Myc* (Cai et al., 2018). qRT-PCR analysis showed that multiple YAP targets in females, including these and *Fxyd1*, were upregulated in the RC/RC vs. WT mice and, as with *Myc,* were downregulated in the RC/RC:*Casp1*KO vs. RC/RC mice (Figures 6A–D). In males, two of the YAP targets, C*tgf* and *Axl* were assessed and while there was an uptrend in these targets in RC/RC vs. WT mice, there was no effect on the levels of these target transcripts in the RC/RC:*Casp1*KO vs. RC/RC mice (Supplementary Figure S6A,B). Assessment of YAP protein in female mouse kidneys showed elevated levels of this protein in RC/RC compared to WT and reduced levels (2 out of 3 cases) in the RC/RC:*Casp1*KO vs. RC/RC kidneys (Figure 6E). Collectively these results suggest that deficiency of Caspase-1 in this PKD model restrains the full activation of both MYC and YAP pathways in females but not males, correlating with the female-specific effects of this deficiency in restraining cystic disease progression.

**FIGURE 7:**
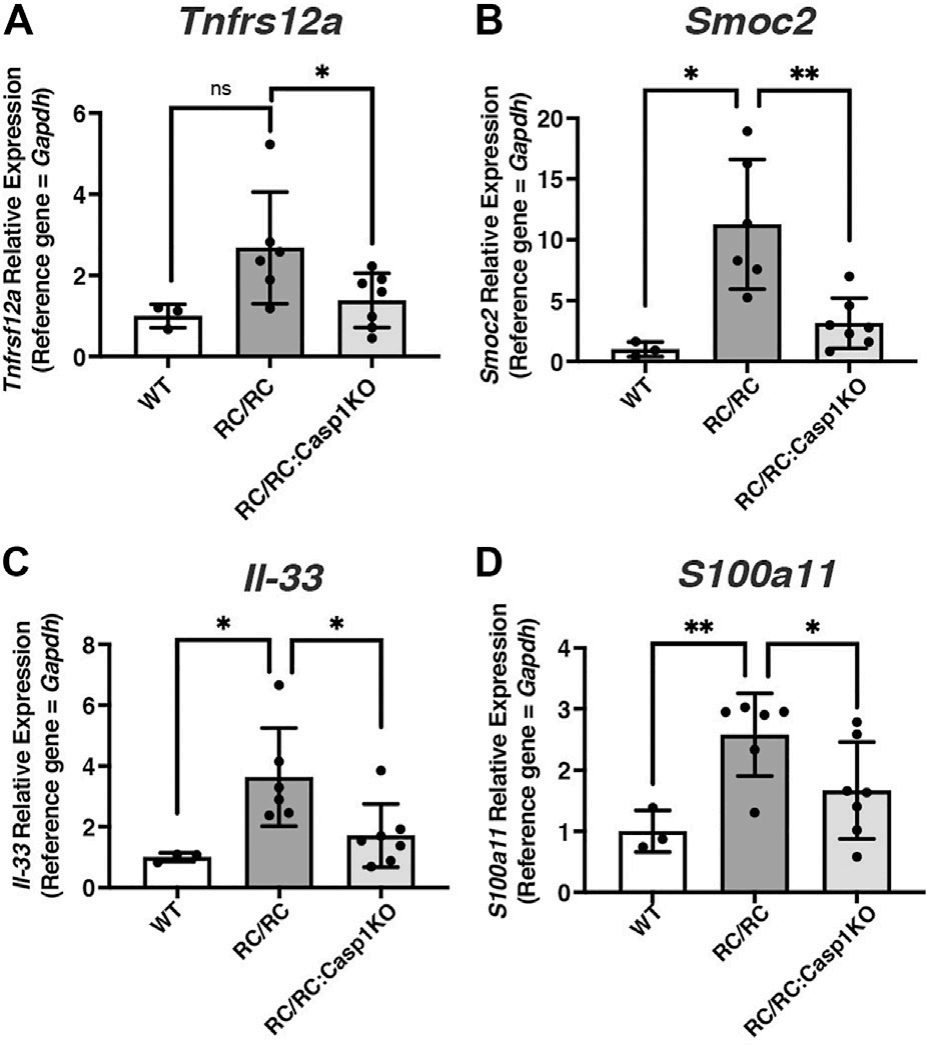
The expression of coordinately regulated YAP/MYC targets upregulated RC/RC female mouse kidneys is diminished by *Casp1* KO. **(A–D)** qRT-PCR of YAP/MYC coordinately regulated transcript targets from WT, RC/RC, and RC/RC:*Casp1*KO female mouse kidneys: **(A)** *Tnfrs12a*; **(B)** *Smoc2*; **(C)** *Il-33;* and **(D)** *S100a11.*

In a recent study YAP was found to activate MYC-dependent transcription cooperatively to promote a full proliferative response through integration of both mitogenic and mechanical signals (Croci et al., 2017). The differentially expressed genes that responded to MYC and YAP together but not, or less so, to either alone were identified and were linked mainly to cell proliferation. Given the known contribution of both MYC and YAP to PKD disease progression and the elevated levels of these proteins in cystic tubular cells (Happe et al., 2011; Trudel, 2015; Cai et al., 2018), we explored the possibility that YAP/MYC transcriptional cooperation may be occurring in PKD kidneys, especially in females, and that inflammasome activation may influence this phenomenon. Expression of these known coordinately regulated genes was assessed in our dataset from WT, RC/RC, and RC/RC:*Casp-1* KO, kidneys. This analysis revealed 65 coordinately YAP/MYC-regulated targets that were upregulated in RC/RC vs. WT and downregulated in RC/RC:*Casp1*KO vs. RC/RC (Supplementary Table S6). Validation of this observation was carried out, initially in females, by qRT-PCR for several of these genes, including those encoding TNF receptor super family member 12A (*Tnfrs12a*), SPARC related modular calcium binding 2 (*Smoc2*), Interleukin 33 (*Il33*), and S100 calcium binding protein A11 (*S100a11*) (Figures 7A–D). In males, while there was an uptrend in the single coordinate YAP/MYC target analyzed in RC/RC vs. WT mice, *Tnfrs12a*, there was no effect on the levels of this target transcript in the RC/RC:*Casp1*KO vs. RC/RC mice as seen in females (Supplementary Figure S6C). Other YAP/MYC-regulated targets were not assessed in males.

Collectively, these results suggest that deficiency of *Caspase-1* in the RC/RC PKD model restrains the activation of both MYC and YAP pathways and likely their coordinately regulated pathways in females but not males. Given the demonstrated critical roles of the YAP and MYC pathways to PKD cystogenesis, it is possible that the restraint of these pathways in female RC/RC mice deficient for *Caspase-1* contributes to the female-specific restraint of cystic disease progression in these mice.

### Hydroxychloroquine constrains inflammasome activation and restrains cystic disease progression in RC mice

Given the encouraging data showing ameliorative effects of *Casp1* depletion in PKD, at least in females, we sought to identify FDA-approved medications with the ability to inhibit inflammasome activation that could potentially be used for long-term treatment in PKD patients. Hydroxychloroquine (HCQ) is widely used to treat chronic inflammatory illnesses, such as lupus and rheumatoid arthritis (Ponticelli and Moroni, 2017; Schrezenmeier and Dorner, 2020), and is low-cost with uncommon side effects. While the mechanisms of HCQ’s immunomodulatory effects are incompletely understood, in studies of the well-known anti-inflammatory effects of HCQ, this drug was shown to dampen ATP-induced inflammasome activation *in vitro* and *in vivo* (Eugenia Schroeder et al., 2017). eATP is likely to be a relevant NLRP3 inflammasome-activating DAMP in PKD because: 1) as we show here, renal expression of the NLRP3 sensor appears to be a common PKD feature, suggesting that priming of this inflammasome has occurred; and 2) abundantly elevated levels of eATP are produced by tubular epithelial cells from PKD kidneys compared to control cells, giving rise to the elevated levels in these diseased kidneys (Wilson et al., 1999; Schwiebert et al., 2002; Palygin et al., 2018). Thus, it seemed reasonable to hypothesize that treatment of PKD mice with HCQ, an inhibitor of ATP-dependent inflammasome activation, might restrain cystic disease progression, similar to our findings in the Caspase-1 KO RC/RC female mice.

Before we tested this hypothesis, we examined the effects of HCQ on inflammasome production of IL-1β and IL-18 in cell-based experiments. In LPS-primed human THP-1 monocytes, HCQ diminished the ATP-induced release of both IL-1β and IL-18 in a concentration dependent manner (Supplementary Figure S7A,B). Similarly, using LPS-treated mouse primary spleen cells, HCQ diminished the ATP-induced release of IL-1β (Supplementary Figure S7C,D). These data support previous findings in the literature and the authors’ conclusions regarding the inhibitory effects of HCQ on inflammasome activation (Eugenia Schroeder et al., 2017).

To test the effects of HCQ in PKD, we treated RC/RC mice (Balb/C background) with HCQ dissolved in the drinking water. The RC/RC mice in the Balb/C background are amenable to assessing the efficacy of short-term treatment regimens on renal function, which is why they were chosen for this study. The Balb/C RC/RC mice begin to show reduced kidney function, evidenced by elevated BUN, earlier than the C57Bl/6 RC/RC mice (typically within a few months) (Arroyo et al., 2021). However, one disadvantage of the cystic disease progressing earlier in the Balb/C RC/RC mice is that, after weaning, changes in the 2K/TBW in the following few months are minimal in the Balb/C RC/RC mice. This reduces the usefulness of this measurement as a parameter of PKD progression in short-term studies begun after weaning.

**FIGURE 8:**
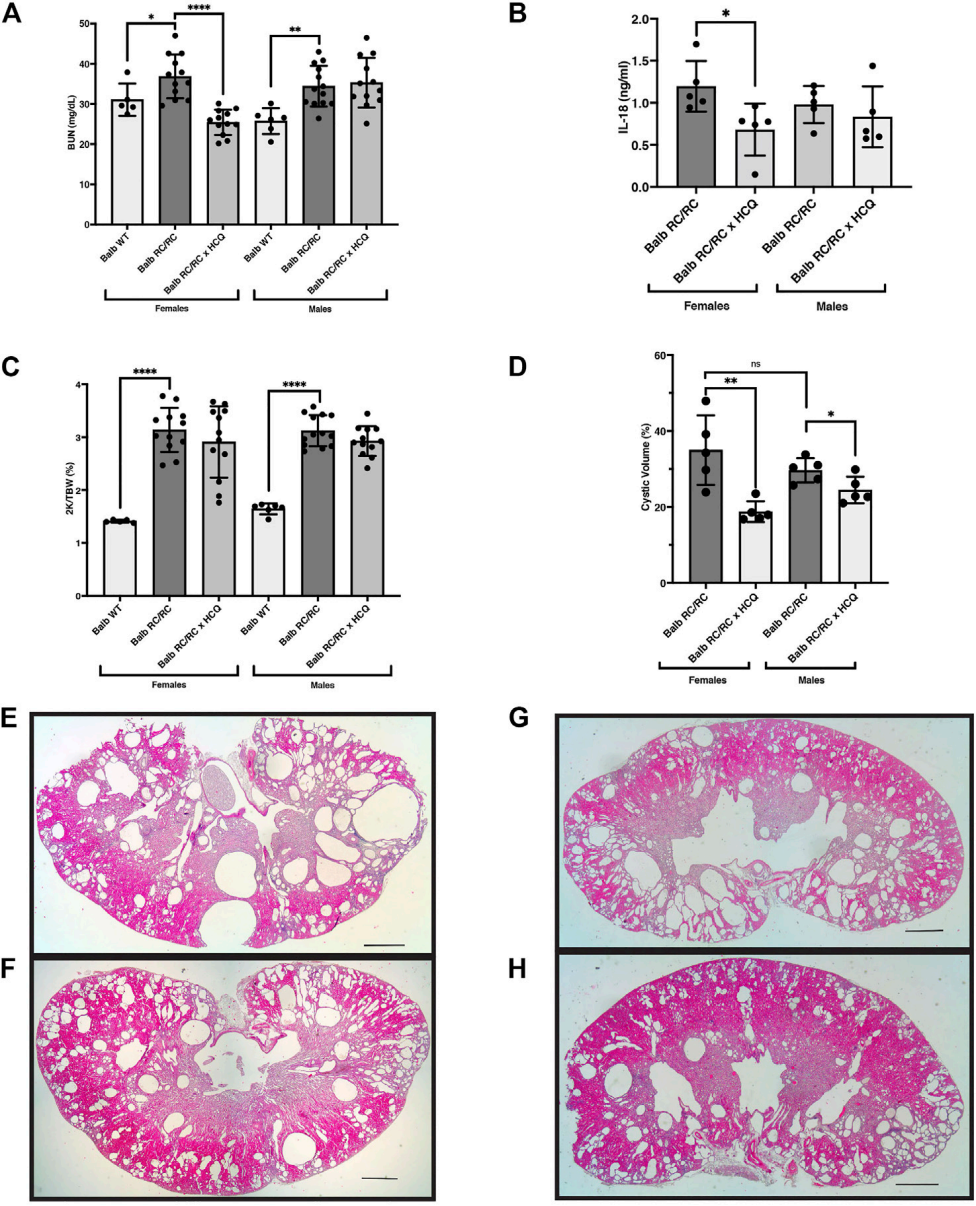
Hydroxychloroquine treatment of RC/RC mice protects kidney function in females and restrains cyst growth in females and males. Shown are measured parameters of PKD progression and serum from RC/RC mice on the BALB/c background that were treated with hydroxychloroquine, and control untreated mice of the same age. **(A)** Blood urea nitrogen (BUN) was determined for each mouse and plotted for females and males. **(B)** Serum IL-18 was measured for the five mice with BUNs closest to the mean for each RC/RC sample type and plotted for females and males. **(C)** The two-kidney/total body weight percentage (2K/TBW %) was determined for each mouse and plotted for females and males. **(D)** The cystic volume as a percentage of total kidney volume was determined for the five mice with BUNs closest to the mean for each RC/RC sample type and plotted for females and males. **(E,F)** Formalin-fixed average sized kidneys from HCQ-treated and untreated RC/RC mice were sectioned and stained with hematoxylin and eosin. **(E)** Untreated female. **(F)** HCQ-treated female. **(G)** Untreated male. **(H)** HCQ-treated male. Scale bars = 1 mm.

With this limitation in mind, we examined the effects of HCQ provided in the drinking water from the time of weaning until 4 months of age. To assess renal function, serum BUN in Balb/C RC/RC control mice and those treated with HCQ was measured. As shown in Figure 8A, HCQ treatment resulted in a significant reduction in BUN in the females, whereas there was no difference in this parameter in males, similar to the observed gender-specific effects on cystic index of *Casp1* KO. To confirm the inhibitory effects of HCQ on inflammasome activation in these mice we measured the serum-levels of IL-18 in five RC/RC mice in both treated and untreated groups having BUNs closest to the mean of each group. Similar levels of this cytokine were found in untreated male and female RC/RC mice. In the HCQ-treated mice, there was significant reduction of this cytokine in the sera of treated females relative to untreated females, whereas in males there was a downward trend of IL-18 levels in the treated group, but it did not reach significance (Figure 8B). As expected, there was no difference in the 2K/TBW in HCQ-treated *versus* untreated mice for either females or males (Figure 8C). To determine the relationship between the protective effects of HCQ and cystic disease, measurements of kidney cystic volumes were carried out. Interestingly, there was a significant reduction of cystic volumes in the HCQ-treated vs. untreated mice in both females and males (Figure 8D), though the effect was especially prominent in females. Stained histological sections of average-sized kidneys corroborated these data, as both females and males showed robust cystic disease in the untreated mice that appeared to be restrained in the HCQ-treated mice (Figures 8E–H).

## Discussion

The inflammasome is a key component of the innate immune response, and activation of the Caspase-1/inflammasome has been shown to be common to many processes known to accelerate cyst formation and expansion, including renal IR, exposure to commensal microbes/microbial products, and deposition of renal crystals. Evidence is presented here for the first time that the Caspase-1/inflammasome is primed in PKD kidneys of both humans and mice and is activated in the cystic kidneys of female RC/RC mice, a human-orthologous PKD model mouse strain. Renal CD11c+ cells were identified in these mice as the predominant immune cells demonstrating the hallmarks of Caspase-1 inflammasome activation (cleavage of pro-Caspase-1 and production of extracellular IL-1β). We also show for the first time that knockout of Caspase-1 slows cyst expansion and disease progression specifically in females suggesting a significant sex difference in the inflammatory environment of these mice. Evidence is also provided that the presence of Caspase-1 in female but not male mice supports MYC/YAP pathway activation in the kidney, which has been shown to play a central role in PKD progression (Trudel, 2015; Cai et al., 2018; Kurbegovic and Trudel, 2020). Finally, we show that treatment of RC/RC mice with the anti-inflammatory drug HCQ, which can inhibit the NLRP3/Caspase-1 inflammasome among its effects, both protects kidney function in females and restrains cystic growth in both females and males, suggesting its potential use in the treatment of ADPKD patients.

In initial studies we found that the expression of multiple cellular sensors that act upstream of Caspase-1/inflammasome activation are elevated in cystic kidneys of both human ADPKD and PKD model mice, suggesting that the inflammasome priming process has occurred. The pattern of inflammasome sensor expression was found to be different between human and mouse PKD kidneys. The human ADPKD kidneys examined are end-stage, so the set of DAMPs contained within these kidneys are likely to be more extensive and probably include MAMPs, since microbial products are routinely detected in these kidneys (Miller-Hjelle et al., 1997). The specific set of DAMPs present in the two PKD model mice, *jck* and RC/RC, are also likely to be different. While these mice are exposed to the same external conditions (identical housing and in the same room), the stage of the cystic disease progression at the time of evaluation for these experiments is not identical, so differences in the number of injured or dying cells releasing DAMPs are likely. In addition, there may be genetic differences influencing the expression of specific damage patterns found in these mice. Elevation of the NLRP3 sensor expression is a common feature among the PKD kidneys (human and mouse) included in this study. This feature, along with the known elevated levels of extracellular ATP in PKD kidneys (Wilson et al., 1999; Schwiebert et al., 2002), suggests that activation of this inflammasome is a likely to be a common feature also.

Within the cystic kidneys of female RC/RC mice, immune cells, particularly CD11c+ cells, were found to be the primary cell showing evidence of inflammasome activation. CD11c+ (also known as Integrin alpha X, *Itgax*) is a cell surface marker found in populations of both macrophages and dendritic cells, so it is likely that inflammasome activation was present in both of these populations, and that they contributed to the Caspase-1-dependent pro-cystic functions in these mice. Since renal CD11c+ cells can be depleted by clodronate liposomes (Mulay et al., 2013; Gottschalk and Kurts, 2015), it is likely that cells expressing activated Caspase-1/inflammasomes were depleted in earlier studies using this drug to suppress cystic disease in PKD mice (Karihaloo et al., 2011; Swenson-Fields et al., 2013; Yang et al., 2018).

Transcriptome analysis of cystic kidneys by RNAseq in this study suggests a potential mechanistic connection between the Caspase-1/inflammasome and cystic disease. Namely, the inflammasome appears to promote YAP/MYC pathways in females specifically. This possibility was supported further by immunoblots of renal YAP and MYC and qRT-PCR of their individual and coordinately regulated target genes. Both of these transcription factors have been previously implicated in PKD pathogenesis. Since the initial discovery of elevated renal MYC levels in a non-orthologous model of PKD (Cowley et al., 1987), this protein and MYC-stimulated pathways have been found to be elevated in all models of PKD that have been examined, as well as in human ADPKD (Trudel, 2015; Kurbegovic and Trudel, 2020). Overexpression of *Myc* in mouse kidneys is sufficient to induce tubular cell proliferation and cystogenesis (Trudel et al., 1991), and genetic deficiency of renal *Myc* in a human-orthologous mouse model of PKD dramatically restrained cyst formation (Cai et al., 2018). *Myc* is also a transcriptional target of YAP, which appears to promote the transcription of *Myc* directly (Cai et al., 2018). Elevated levels of YAP and YAP target genes have been found in human ADPKD kidneys and in multiple orthologous mouse models of PKD (Happe et al., 2011). Genetic deficiency of YAP and its transcriptional coactivator, TAZ, reduced Myc expression and suppressed cyst formation in *Pkd1*-deficient mouse kidneys (Cai et al., 2018). In these studies, a pathway flowing from YAP to MYC was shown to be an important contributor to cystogenesis in the *Pkd1* mutant mouse kidneys.

Crosstalk between innate immune pathways and the Hippo-YAP pathway have been documented previously. The release of inflammatory cytokines, including TNFα, IL-6, and most notably, the inflammasome-generated cytokine IL-1β (Taniguchi et al., 2015; Liu et al., 2020; Wang et al., 2020; Caire et al., 2022), have been shown to stabilize levels of YAP protein and activate its target transcriptional pathways. The effects of activated YAP appear to be cell-type specific. In tubular epithelial cells, this pathway typically results in cell proliferation and contributes to regeneration of epithelial cells after injury (Taniguchi et al., 2015; Xu et al., 2016; Chen et al., 2018). Activation of YAP in mononuclear immune cells by inflammatory cytokines appears to promote inflammatory pathways further, including the upregulation of inflammatory cytokines/chemokines (Liu et al., 2020; Caire et al., 2022). While it seems likely that the reduced renal levels of YAP and YAP target genes, including *Myc,* that we show here in female RC/RC:*Casp1* KO mice contribute to their suppressed cystic disease, the specific contribution of this pathway to direct effects on tubule cell proliferation or to the inflammatory environment influencing cyst growth has not yet been studied.

In the studies reported here, knockout of *Casp1* slowed cyst expansion and disease progression, but the effects were statistically significant only in females. There were no apparent differences in disease severity between males and females. The reason for the gender-specific effects of *Casp1* deletion is uncertain. It is possible that there are female-specific influences on inflammasome activation. In humans, the baseline expression of multiple Caspase-1 inflammasome sensors, including *NLRP1*, *NLRC4,* and *MEFV*, was found to be elevated in macrophages derived from peripheral blood mononuclear cells of healthy females compared to males, suggesting that estrogen might play a role in promoting macrophage inflammasome priming and activation (Yang et al., 2015). Such an effect might contribute to the sex differences found in the incidence of those autoinflammatory conditions that occur primarily in females and to which the inflammasome contributes, including rheumatoid arthritis, systemic lupus erythematosus, Sjogren’s syndrome, and multiple sclerosis (Orstavik, 2017; Govindarajan et al., 2020; Li et al., 2020). In contrast to these diseases, however, cystic disease in human ADPKD and in multiple PKD mouse models is not found primarily in females and is typically worse in males compared with females (Schrier et al., 2014). Regardless, a female-specific upregulation of multiple Caspase-1 inflammasome sensors in the immune cells of the RC/RC mice could sensitize these cells to Caspase-1/inflammasome activation.

It is also possible that males exhibit a dominance of Caspase-1-independent, inflammatory pathways. This hypothesis is supported by the results of treatment with HCQ (Figure 8). Previous studies have shown that HCQ can inhibit the NLRP3 inflammasome in immune cells (Eugenia Schroeder et al., 2017; Tang et al., 2018), and our data support these results (Supplementary Figure S7). Males and female RC/RC mice have similar, elevated levels of serum IL-18, however, HCQ lowered serum levels of IL-18 in females but not significantly in males. In males the production of this cytokine is likely occurring *via* non-canonical inflammatory mechanisms (Afonina et al., 2015; Netea et al., 2015). Such Caspase-1-independent inflammatory pathways in RC/RC males may be related to the reported amplified response to renal ischemic injury that occurs in males (Kher et al., 2005), which could be relevant in PKD because of the ongoing ischemic and mechanical injury arising from vascular compression and cyst expansion (Grantham et al., 2011). Thus, an amplified, non-canonical inflammatory response predominating in male RC/RC mice, coupled with inflammasome sensitization in females, could reasonably account for the female-dominant ameliorative effects of *Casp1* KO.

HCQ treatment also had gender-specific effects on the RC/RC mice, i.e., female-specific protection of kidney function that correlated with female-specific reduced serum levels of IL-18. However, HCQ significantly restrained cyst expansion in both males and females, though the effect was more robust in females. The minimal effects on serum IL-18 levels in HCQ-treated males in this study suggest that the restrained cystic expansion in HCQ-treated males is arising from other HCQ effects outside of those on inflammasome activation.

HCQ is an anti-malarial agent, which is also commonly used to treat multiple autoinflammatory diseases, including those to which the NLRP3 inflammasome is known to contribute: rheumatoid arthritis, systemic lupus erythematosus, and Sjögren’s syndrome (Nirk et al., 2020). The drug has multiple cellular effects in addition to NLRP3 inflammasome inhibition that are likely to influence the inflammatory environment and could affect PKD progression (Nirk et al., 2020). The mechanism of inhibition of ATP-induced NLRP3 inflammasome by HCQ in THP-1 macrophages was shown to due to the inhibition of Ca^2+^-activated K^+^ channels, including the KCa3.1 (KCNN4) channel, and consequent inhibition of K^+^ efflux (Eugenia Schroeder et al., 2017). Notably, the KCa3.1 channel is also expressed in tubular epithelial cells and was shown to play an important role in cAMP-dependent chloride secretion and cyst growth in *vitro* studies of ADPKD cyst cells (Albaqumi et al., 2008). Similar HCQ inhibition of this channel *in vivo* could restrain fluid secretion and cyst growth in PKD mice, in addition to its inhibitory effects on the NLRP3 inflammasome. Also, HCQ is known to impair endosome acidification, and, since multiple TLRs require acidified endosomes for their activation, this effect results in the impairment of TLR-mediated production of cytokines, including TNFα, IL-6, and IFN-γ (Nirk et al., 2020). Actions of HCQ such as these could be responsible for the HCQ-mediated suppression of cyst growth in male RC/RC mice, and they could contribute to the effects in females. Regardless of the gender-specific effects of HCQ treatment on PKD progression in these mice, the reduction in cystic volume of both sexes in this study suggests that HCQ might be effective in the treatment of patients with ADPKD.

In sum, we have provided evidence that the Caspase-1/inflammasome is activated during the course of PKD in RC/RC mice, is an important driver of PKD progression in females and may be a viable therapeutic target. Several relevant questions remain. Is Caspase-1/inflammasome activation a common feature of all renal injuries that promote polycystic kidney disease? The answer is uncertain. Treatment of PKD mice with the nephrotoxin 1,2-dichlorovinyl-cysteine (DCVC) to induce renal injury accelerates cyst formation significantly (Happe et al., 2009). However, effects of this nephrotoxin on Caspase-1/inflammasome activation have not been examined.

Another question to be explored is whether renal activation of Caspase-1/inflammasome in PKD mice always leads to accelerated cyst formation and disease progression. Also, it is worth considering whether activated Caspase**-**1 is sufficient to promote *de novo* cyst formation in the absence of mutations that cause PKD. Such a scenario is suggested by the studies of (Kurbegovic and Trudel, 2016), who showed that renal IR, a Caspase-1-activating process, was sufficient to promote *de novo* cyst formation in WT mice. Additionally, it was recently uncovered that patients with either hereditary hypophosphatemic rickets with hypercalciuria or CYP24A1 deficiency show a high incidence of renal cysts (Hanna et al., 2021; Hanna et al., 2022). A characteristic feature of these disorders is renal stones, which would suggest the likely presence of activated Caspase-1. It may be that the pro-cystic effects of activated Caspase-1 demonstrated in this paper may also be present in the absence of mutations that cause PKD. Experiments to test this possibility are currently underway.

## Materials and methods

An expanded version of the methods is included in Supplementary Materials and Methods. Detailed methods for production of the RC/RC:*Casp1KO* mouse, HCQ treatment of RC/RC mice*,* histology, immunohistochemistry, quantitative RT-PCR, determination of cystic volume, preparation and isolation of primary immune cells, flow cytometry, and cell culture are included. All animal experiments were approved by KUMC Institutional Animal Care and Use Committee, and the use of human tissue was approved by the KUMC Institutional Review Board. Data are presented as the mean ^±^ s.d. and were compared using the two-tailed *t*-test calculated with Prism (v9.2, GraphPad, La Jolla, CA). The *p*-values of <0.05 were considered significant, as indicated in the figures.

## Data Availability

The datasets presented in this study can be found in online repositories. The names of the repository/repositories and accession number(s) can be found below: https://www.ncbi.nlm.nih.gov/geo/, GSE207957.
