# Supplementary material for: Caspase-1 and the inflammasome promote polycystic kidney disease progression
Source: Front Mol Biosci. 2022 Nov 29;9:971219. doi: 10.3389/fmolb.2022.971219 (PMC9745047; doi:10.3389/fmolb.2022.971219)
Supplement: Supplementary file 1 [file DataSheet2.PDF]

Supplementary Figure 1

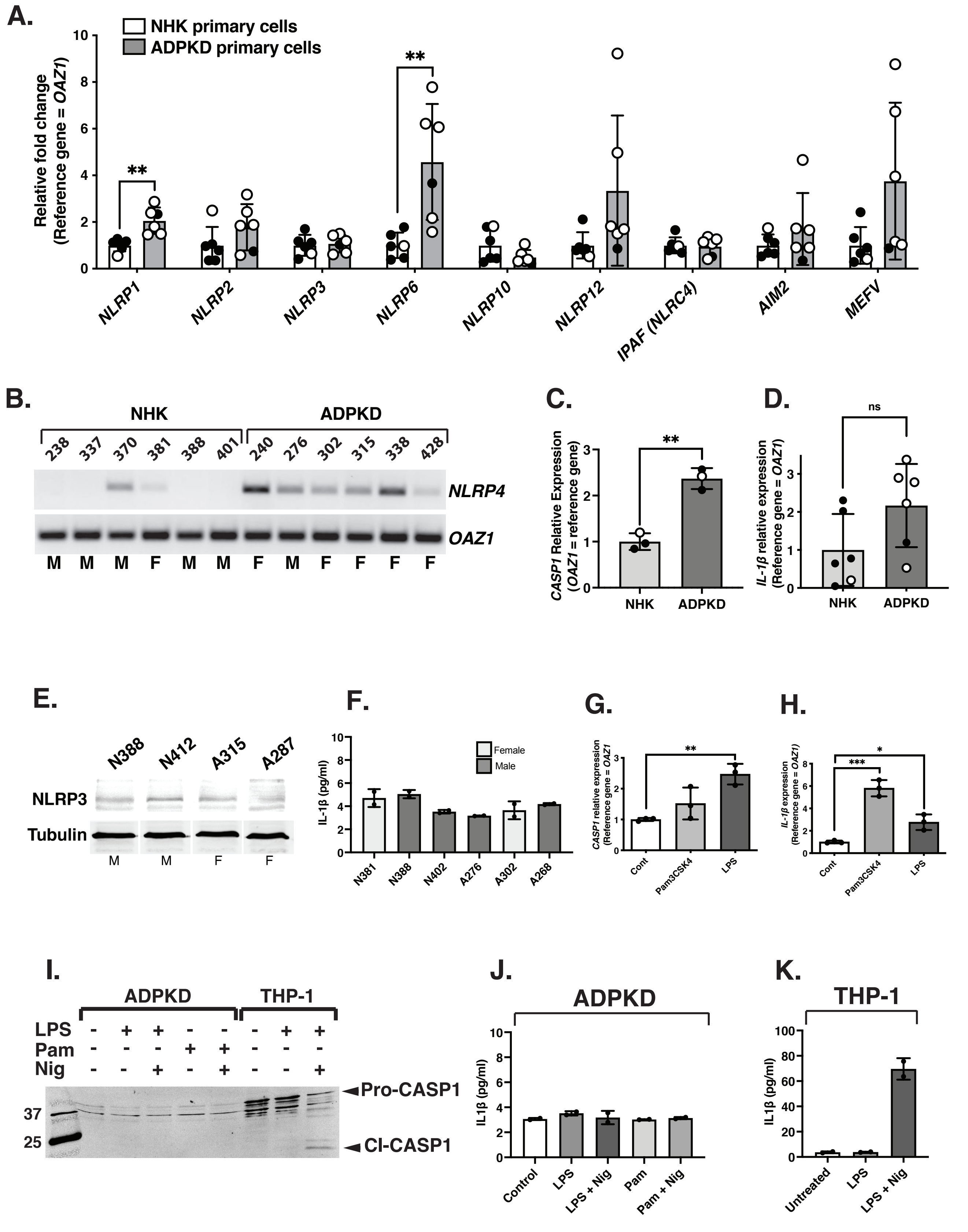

S1. Absence of induced inflammasome activation in primed human ADPKD renal cyst epithelial cells. (**A)** qRT-PCR of transcripts encoding inflammasome sensors from primary tubular cells isolated from NHK and cysts from ADPKD patients showing upregulation of NLRP1 and NLRP6 (**p<0.01). Open circles = females; closed circles = males. (**B)** Semi-quantitative PCR of *NLRP4* and reference gene (*OAZ1*) from cDNAs of transcripts from the same primary tubular cell samples as in (**A**). Assigned kidney numbers and sex are shown for each NHK and ADPKD cell sample. (**C and D)** qRT-PCR of transcripts encoding Caspase-1 (**C)** and IL-1β (**D)** from ADPKD and NHK primary tubular cell samples. Symbols indicating sex are the same as in (**A**). (**E)** Western blot of NLRP3 and tubulin from primary NHK and ADPKD cyst cell protein samples. Assigned kidney numbers and sex are shown. Intervening lanes on blot have been deleted. **(F)** Concentrations of IL-1*β* in duplicate samples of condition media collected from primary NHK and ADPKD cyst cells after being cultured for 24 h and measured by ELISA. Assigned kidney numbers and sex are shown. **(G and H)** Primary cyst cells from ADPKD patients (all males). were treated with Pam3CSK4 (a synthetic mimic of bacterial lipopeptides and a TLR 1/2 agonist) or LPS (a TLR 2/4 agonist) or were left untreated. qRT-PCR of transcripts encoding Caspase-1 (**G**) and IL-1β (**H**) from these cells was performed to evaluate whether the TLR agonist treatments were sufficient to induce inflammasome priming (*p<0.05; **p<0.01; ***p<0.001). (**I**) Primary ADPKD cyst cells or THP-1 monocytes were treated with either LPS or Pam3CSK4 (Pam) followed by the addition of a potassium ionophore, nigericin (Nig). Proteins from these cell samples were analyzed by Western blot using anti-Caspase-1 antibody. The conditioned media from these cells were collected, and the levels of IL-1β were measured by ELISA and the results plotted separately for ADPKD cyst cells (**J**) and THP-1 monocytes (**K**). This experiment was repeated using primary ADPKD cyst cells from 2 additional patients with similar results. The THP-1 cells treated with TLR agonist and nigericin showed both Caspase-1 cleavage and release of IL-1β into the conditioned media, indicating inflammasome activation, whereas ADPKD renal cyst epithelial cells treated similarly showed neither effect.

Supplementary Figure 2

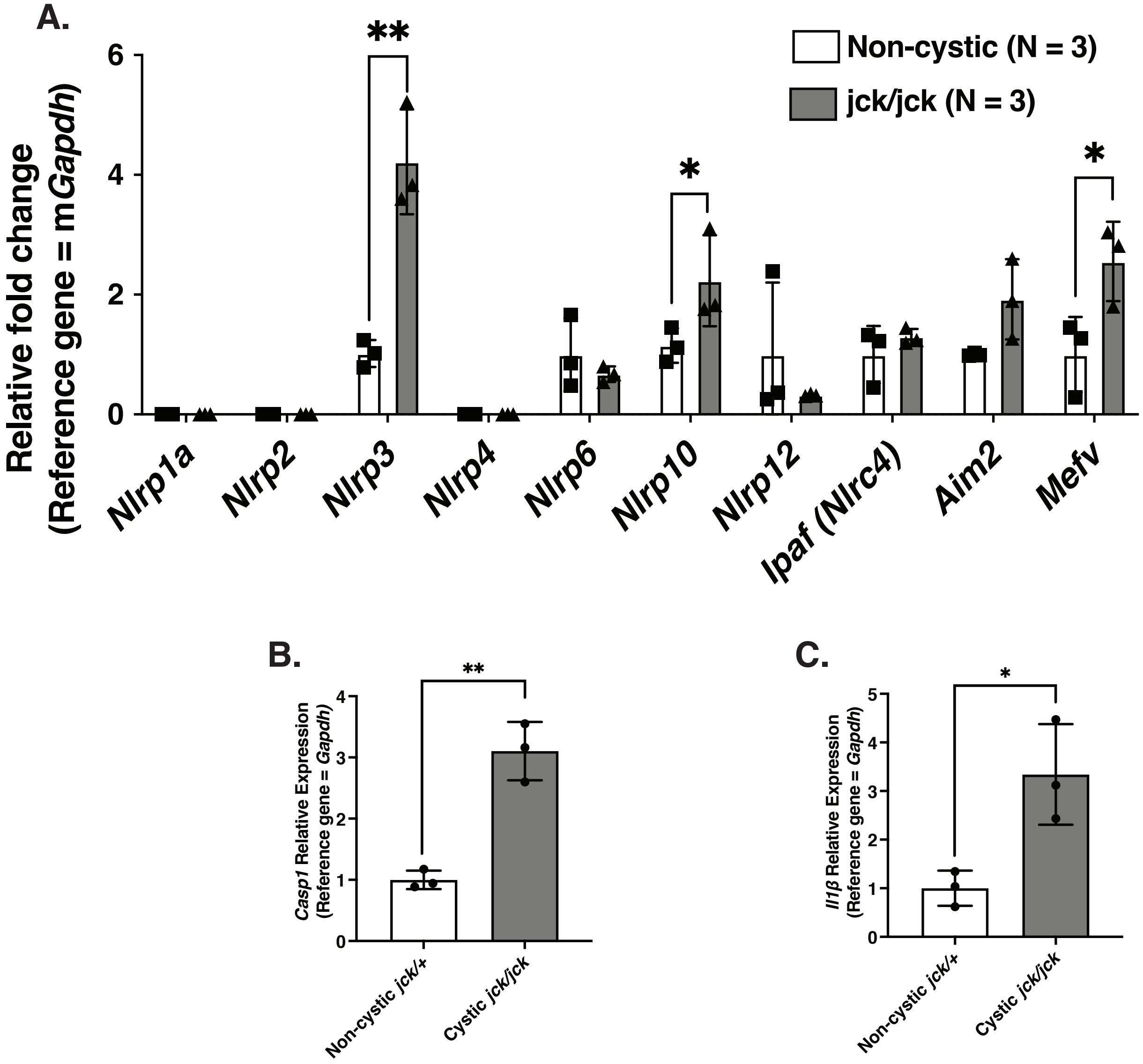

S2. Inflammasome components are elevated in kidneys from *jck* mice. (**A)** qRT-PCR of transcripts encoding inflammasome sensors from kidneys of cystic (*jck/jck*) or non-cystic (*jck/+*) mice on PN38 (all males). qRT-PCR of transcripts encoding Caspase-1 (**B**) and IL-1β (**C**) from cystic (*jck/jck*) or non-cystic (*jck/+*) kidney tissues samples.

Supplementary Figure 3

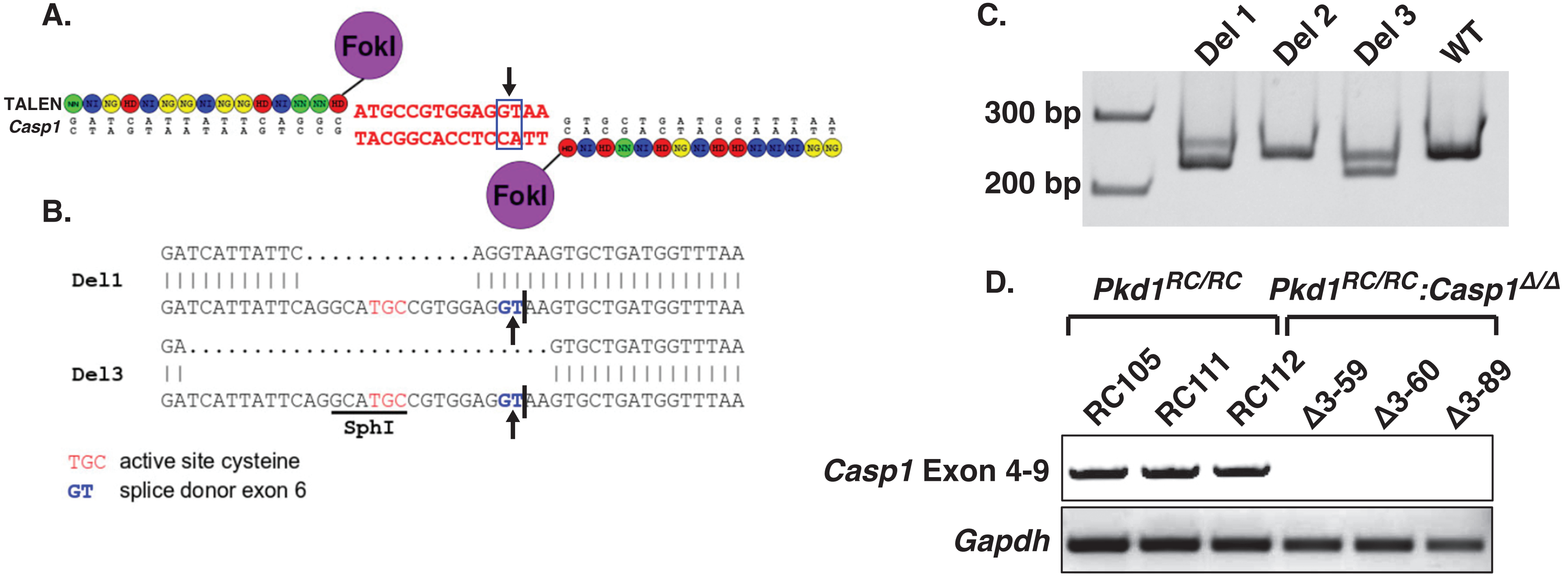

S3. Targeted disruption of murine *Casp1* gene mice using TALENs. (**A)** Targeting of the 3' end of exon 6 of the *Casp1* gene on mouse chromosome 9. Each TALEN targeted 15 bp of the *Casp1* gene with a 15 bp gap (shown in red letters) that included the splice donor of exon 6 (arrow). Colored circles denote the repeat-variable di-residue within the TALENs, with the specific recognized nucleotide in *Casp1* shown below. Fok1 = Fok1 endonuclease. (**B**) Shown are the sequences of two deletions generated in the *Casp1* gene. Those mice with Del3, which have a deletion of 28 bp that includes the coding region for the active site cysteine residue of Caspase-1 (in red letters), the splice donor of exon 6 (blue letters indicated by arrows), and the endonuclease restriction site Sph1 were chosen for propagation and breeding. (**C**) PCR of *Casp1* gene in TALENs-mutated and WT pups. Primers flanking the TALENs-targeted site in the *Casp1* gene were used for PCR. Shown are the 248 bp product from WT mouse DNA and the products generated from pups following TALENs mutation showing the presence of deletions in Del1 and Del3. (**D**) Semi-quantitative PCR of cDNAs made with total RNA isolated from either RC/RC mice with intact *Casp1* ($Pkd1^{RC/RC}$) or RC/RC mice containing a homozygous deletion (Del3 in **B**) in the *Casp-1* gene ($Pkd1^{RC/RC}$:$Casp1^{\Delta/\Delta}$) using primers flanking exons 4-9. PCR products of *Gapdh* generated from the same cDNA samples are also shown for reference.

Supplementary Figure 4

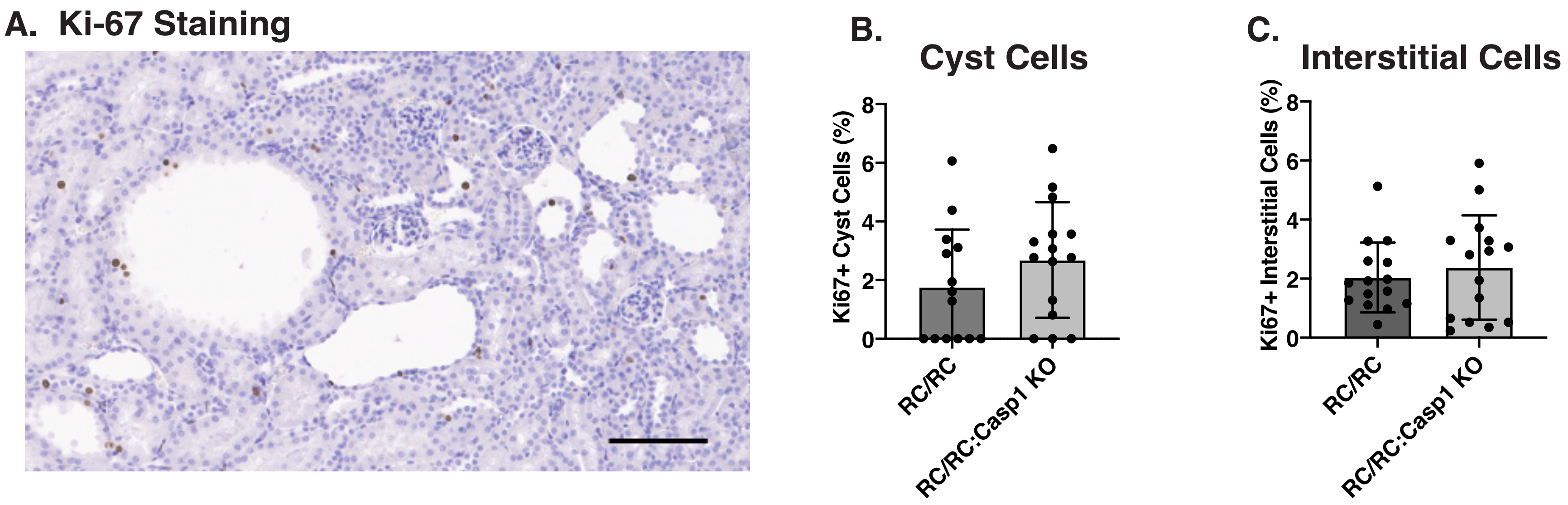

S4. Ki-67 staining from RC/RC and RC/RC-*Casp1*KO female mice. Formalin-fixed sections from RC/RC and RC/RC-*Casp1*KO female mice (3 each) having 2K/TBW % that were closest to the mean shown in Figure 4B were stained with an antibody to Ki-67. (**A)** A representative image from a RC/RC-*Casp1*KO mouse is shown. Scale bar = 100µm. **(B)** Ki-67+ cells per total number of cyst lining cells (%) in each field counted from RC/RC and RC/RC-*Casp1*KO mice. (**C)** Ki-67+ cells per total number of interstitial cells (%) in each field of the same mice as in (**B).**

Supplementary Figure 5

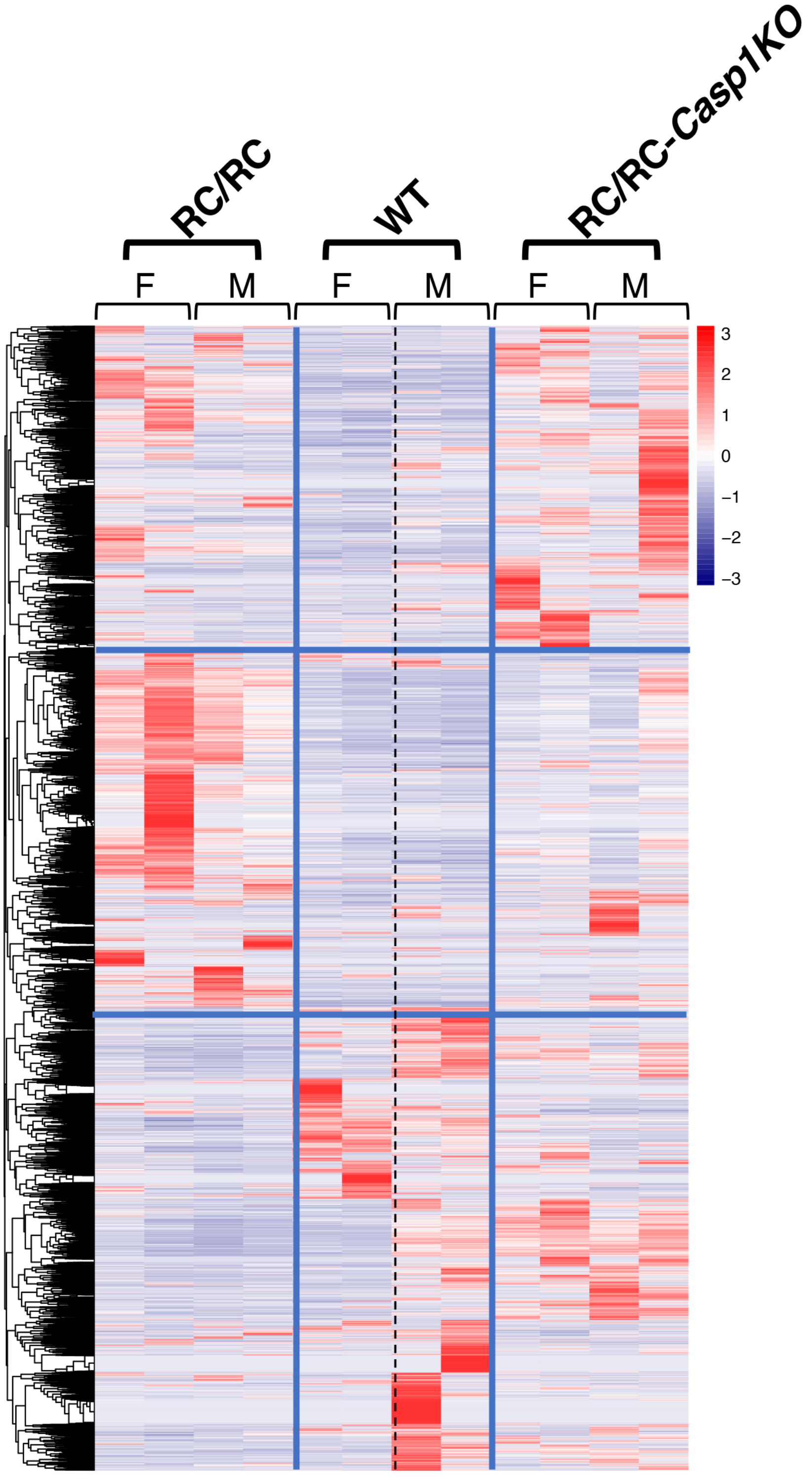

S5. Heatmap showing the normalized expression counts of individual samples. The raw counts were normalized using the R package DeSeq2 (Love et al., 2014). The heatmap shows the normalized expression of a subset of 10,000 genes. These were selected as follows. Two differential expression analyses (WT v RC) and (WT v KO) were performed and for each gene, the average of the absolute log-fold change values was calculated. Only the top 10,000 genes showing the largest absolute deviation were kept and their normalized expression counts are shown in the figure.

Supplementary Figure 6

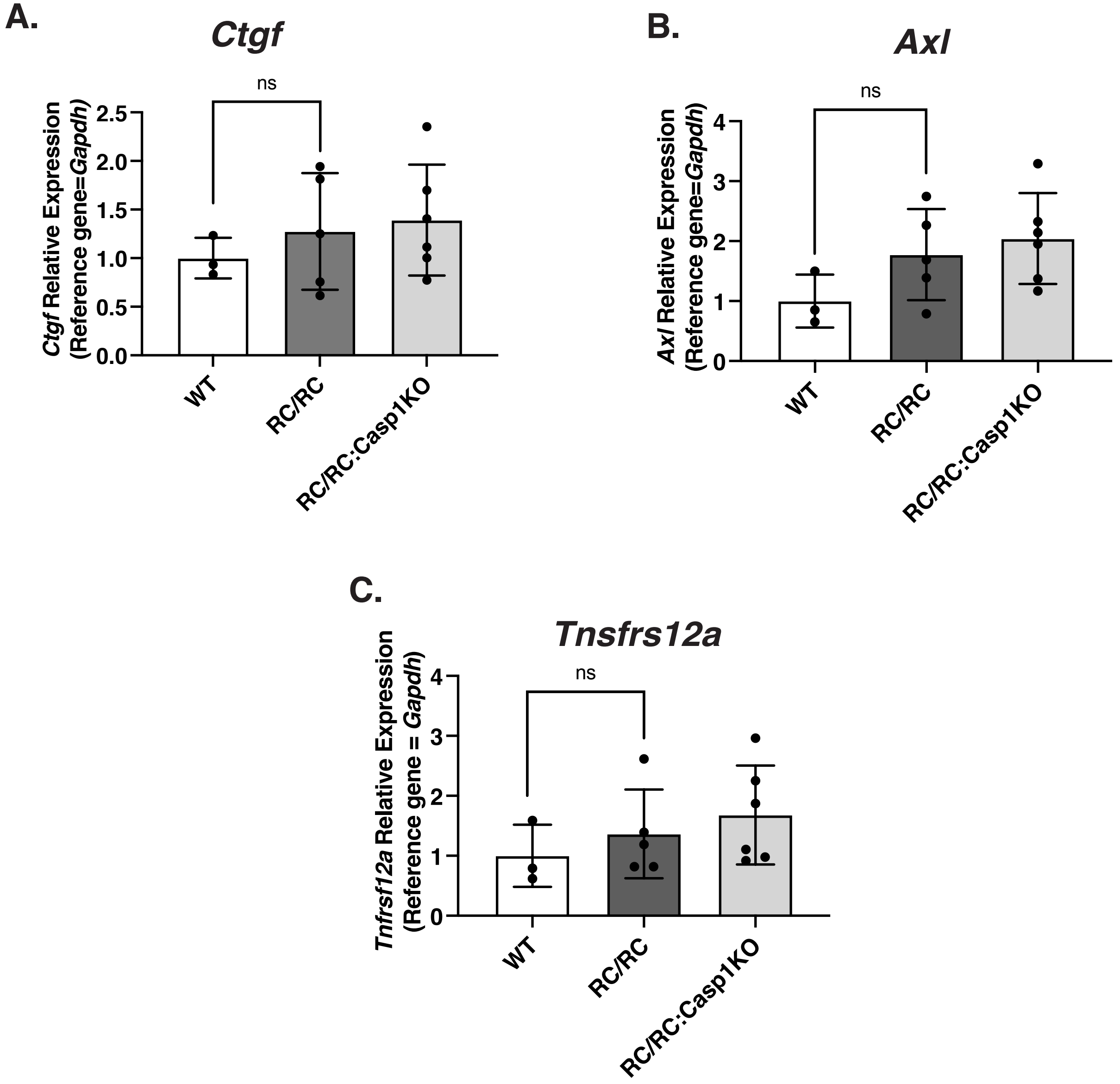

S6. Expression of YAP targets and a coordinately regulated YAP/Myc target in male WT, RC/RC and RC/RC:*Casp-1*KO mouse kidneys. (**A-C**) qRT-PCR of YAP transcript targets (**A-B**) and a coordinately regulated YAP/Myc target (**C**) from WT, RC/RC and RC/RC:*Casp-1*KO male mouse kidneys: (**A**) *Ctgf*; (**B**) *Axl*; (**C**) *Tnfrs12a.*

Supplementary Figure 7

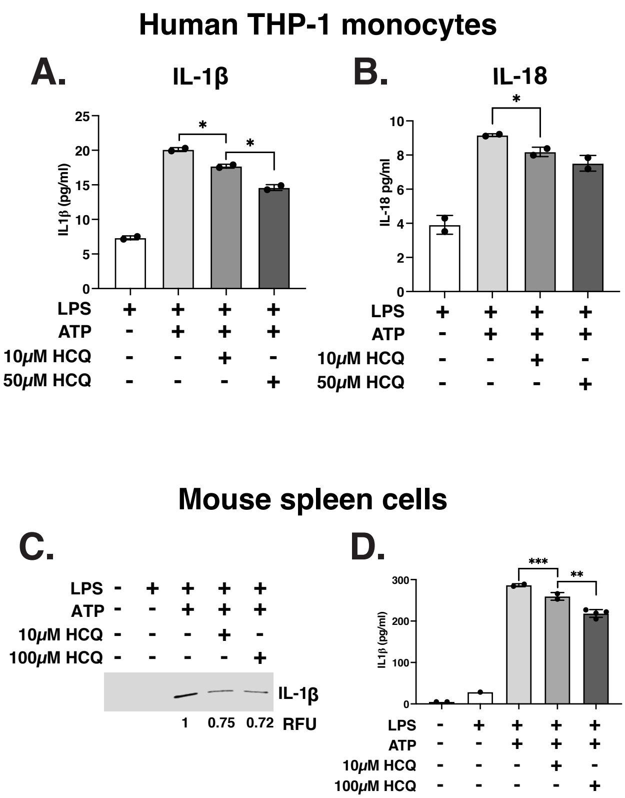

S7. Effects of hydroxychloroquine on ATP-induced inflammasome-activated cytokine release in human THP-1 monocytes and mouse spleen cells. THP-1 monocytes were primed with 100 ng/ml LPS for 3 h and then washed and resuspended in serum-free media. When HCQ was used, it was added at this step for 15 min. ATP (5 mM) or media, to maintain equivalent volumes for all samples, was then added to activate the inflammasome for 45 min prior to collection of the culture supernatants. IL-1β (**A)** and IL-18 (**B)** were quantified in these culture supernatants by ELISA. The results from one experiment representative of three is shown. (**C)** Primary single cells were prepared from a C57BL/6 mouse spleen and incubated overnight in media containing 10% bovine serum to which GM-CSF and M-CSF (5 ng/ml each) had been added. Cells were then removed from the culture dish, washed in PBS, and resuspended in serum-free media. For priming, LPS (1 µg/ml) was added for 3 h. When HCQ was used it was added after 1 h. To activate the inflammasome, ATP (2 mM) was added for 2 h. To maintain equivalent volumes for all samples, serum-free media was added when appropriate. Culture supernatants were collected and the relative levels of IL-1β were assayed by immunoblot (**C**) or were quantified by ELISA (**D).** RFU = relative fluorescence units determined for IL-1β bands following scanning. This experiment was carried out three times with similar results.
