# Supplementary material for: Caspase-1 and the inflammasome promote polycystic kidney disease progression
Source: Front Mol Biosci. 2022 Nov 29;9:971219. doi: 10.3389/fmolb.2022.971219 (PMC9745047; doi:10.3389/fmolb.2022.971219)
Supplement: Supplementary file 3 [file Table1.DOCX]

Supplementary Table 1: Primers used for PCR and qRT-PCR.

|  | **HUMAN** | |
| --- | --- | --- |
| **Gene** | *forward* | *reverse* |
| *OAZ1* | 5'-CACCATGCCGCTCCTAAG-3' | 5'-GAGGGAGACCCTGGAACTCT-3' |
| *NLRP1* | 5'-GCAGTGCTAATGCCCTGGAT-3' | 5'-GAGCTTGGTAGAGGAGTGAGG-3' |
| *NLRP2* | 5'-TGGCCTGGAGATAGCAAAGAG-3' | 5'-CACCACCGTGTATGAGAAGGG-3' |
| *NLRP3* | 5'-GAAGAAAGATTACCGTAAGAAGTACAGAAA-3' | 5'-CGTTTGTTGAGGCTCACACTCT-3' |
| *NLRP4* | 5'-ACACAAAGACCTATCAAGCTCAC-3' | 5'-AAAAGGCGGTCCAAATGGTCA-3' |
| *NLRP6* | 5'-CCTACCAGTTCATCGACCAGA-3' | 5'-CTCAGCAGTCCGAAGAGGAA-3' |
| *NLRP10* | 5'-CTACTTACGGGATATGACCCTGT-3' | 5'-CCAGGTCCACCGGAATCAG-3' |
| *NLRP12* | 5'-GGGGCTTGTCAGGAGATGG-3' | 5'-AGTCCCTGGCATAGTAACCTC-3' |
| *NLRC4* | 5'-TGCATCATTGAAGGGGAATCTG-3' | 5'-GATTGTGCCAGGTATATCCAGG-3' |
| *AIM2* | 5'-TGGCAAAACGTCTTCAGGAGG-3' | 5'-AGCTTGACTTAGTGGCTTTGG-3' |
| *MEFV* | 5'-CTTCCCCGAGGCAGTTTCTG-3' | 5'-CTTCAGGTGGCGCTTACAC-3' |
| *CASP1* | 5'-GCCAAATTTGCATCACATACA-3' | 5'-ATAGCTGGGTTGTCCTGCAC-3' |
| *IL1B* | 5'-ACAGATGAAGTGCTCCTTCCA-3' | 5'-GTCGGAGATTCGTAGCTGGAT-3' |
|  |  |  |
|  | **MOUSE** | |
| **Gene** | *forward* | *reverse* |
| *Gapdh* | 5'-CCACTCACGGCAAATTCAAC-3' | 5'-GTAGACTCCACGACATACTCA-3' |
| *Nlrp1a* | 5'-GGTGGTGTGAAGATGTTGTGT-3' | 5'-TCCATGTTCATCGTAGGGACC-3' |
| *Nlrp2* | 5'-GAAAGCTGGACAAGACTGAGTT-3' | 5'-GGCAGTGGTCTGTGAGAATTT-3' |
| *Nlrp3* | 5'-ATTACCCGCCCGAGAAAGG-3' | 5'-TCGCAGCAAAGATCCACACAG-3' |
| *Nlrp4* | 5'-TGTGATATGTCTGAACGGGAGT-3' | 5'-GAAAGAGGATTTGGGGAGCATC-3' |
| *Nlrp6* | 5'-CTCGCTTGCTAGTGACTACAC-3' | 5'-AGTGCAAACAGCGTCTCGTT-3' |
| *Nlrp10* | 5'-TCAAGACGCTGAAGTTCCACT-3' | 5'-TGCTCCGTACATTGAAATCAGTT-3' |
| *Nlrp12* | 5'-GGATGGCCTCTATCGACTGTC-3' | 5'-CCTCTGCAATCCCCAGGAATAA-3' |
| *Nlrc4* | 5'-ATCGTCATCACCGTGTGGAG-3' | 5'-GCCAGACTCGCCTTCAATCA-3' |
| *Aim2* | 5'-GTCACCAGTTCCTCAGTTGTG-3' | 5'-CACCTCCATTGTCCCTGTTTTAT-3' |
| *Mefv* | 5'-TCATCTGCTAAACACCCTGGA-3' | 5'-GGGATCTTAGAGTGGCCCTCC-3' |
| *Casp1* | 5'CTTTCAAGCTTGGGCACTTC-3' | 5'-CACAGCTCTGGAGATGGTGA-3' |
| *Il1b* | 5'-GCAACTGTTCCTGAACTCAACT-3' | 5'-ATCTTTTGGGGTCCGTCAACT-3' |
| *Myc* | 5'-TCTCCACTCACCAGCACAACTAGG-3' | 5'-ATCTGCTTCAGGACCCT-3' |
| *Ctgf* | 5'-AGTGTGCACTGCCAAAGATG-3' | 5'-CCAGGCAAGTGCATTGGTAT-3' |
| *Axl* | 5'-ATGGCCGACATTGCCAGTG-3' | 5'-CGGTAGTAATCCCCGTTGTAGA-3' |
| *Cyr61* | 5'-GCTCAGTCAGAAGGCAGAC-3' | 5'-GTTCTTGGGGACACAGAGGA-3' |
| *Fxyd1* | 5'-TCCATTCACCTACGATTACCACA-3' | 5'-GAATTTGCATCGACATCTCTTGC-3' |
| *Tnrs12a* | 5'-GTGTTGGGATTCGGCTTGGT-3' | 5'-GTCCATGCACTTGTCGAGGTC-3' |
| *Smoc2* | 5'-AGTGGAGACATTGGCAAGAAG-3' | 5'-ACACACTTTTTGGGCTTGGATT-3' |
| *Il33* | 5'-TCCAACTCCAAGATTTCCCCG-3' | 5'-CATGCAGTAGACATGGCAGAA-3' |
| *S100a11* | 5'-AAGTACAGCGGGAAGGATGGA-3' | 5'-ATGCGGTCAAGGACACCAG-3' |
