# Supplementary material for: Caspase-1 and the inflammasome promote polycystic kidney disease progression
Source: Front Mol Biosci. 2022 Nov 29;9:971219. doi: 10.3389/fmolb.2022.971219 (PMC9745047; doi:10.3389/fmolb.2022.971219)
Supplement: Supplementary file 4 [file DataSheet1.PDF]

Supplementary Materials and Methods

Mice

The $Pkd1^{RC/RC}$ mice (RC/RC) on both C57BL/6J and BALB/c background strains were developed as a knock-in mouse model containing a hypomorphic mutation (R3277C) matching a human ADPKD mutation and provide a slowly progressive model of ADPKD (Hopp et al., 2012). These mice were each maintained on their respective backgrounds in populations containing those that were heterozygous for the mutation. WT, $Pkd1^{+/+}$ of each background type, C57BL/6J (no. 000664, Jackson Laboratory, Bar Harbor, ME) and BALB/c (BALB/cJ, no. 000651, Jackson Laboratory) were also maintained. Genotyping of the $Pkd1^{RC/RC}$ mice was carried out as described (Arroyo et al., 2021) with the initial generation of a PCR fragment (using primers 5'- CAAAGGTCTGGGTGATAACTGGTG-3' and 5'-CAGGACAGCCAAATAGACAGGG-3') followed by digestion with Hinf1 (no. R0155S, New England Biolabs, Ipswich, MA). The *jck* mice, originally from the the lab of David Beier (Atala et al., 1993, Liu et al., 2002), were a gift of Pamela Tran. Genotyping is as described for C57BL/6J-Nek8^{jck}/J (no. 002561, Jackson Laboratory, Bar Harbor, ME).

Generation of TALENs for Casp1 gene and production of the Casp1 knockout mouse

Using the mouse assembly C57BL/6J chromosome 9, GRCm39, we targeted bases 5303749-5303793 at the 3' end of exon 6 in the murine *Casp1* gene. The TALENs were made using the assembly method of Cermak, et al. 2011 (Cermak et al., 2011), a two stage `Golden Gate' assembly cloning method into RCIscript-GoldyTALEN (β-lactamase). RCIscript-GoldyTALEN was a gift from Daniel Carlson & Stephen Ekker at the Mayo Clinic Rochester (Addgene plasmid # 38142). The repeat variable diresidues (RVDs) utilized were TALENS NN NI NG HD NI NG NG NI NG NG HD NI NN NN HD and NG NG NI NI NI HD HD NI NG HD NI NN HD NI HD. mRNA was generated using a T3 message machine kit (no. AM1348, Thermo Fisher Scientific) and purified with a MEGAclear Transcription Clean-Up Kit (no. AM1908, Thermo Fisher Scientific). To generate a knockout mouse, both male and female C57BL/6J (#000664) mice were purchased from Jackson Laboratory (Bar Harbor, ME). Ten female C57BL/6J mice were superovulated at four weeks of age with 5 IU P.G. 600 (Intervet, Summit, NJ) administered intraperitoneally, followed 46-48 h later with 5 IU recombinant hCG (NHPP) and immediate mating to C57BL/6J stud males. Females were sacrificed the morning after mating for collection of oviducts. Dissected oviducts were placed in M2 medium (EMD Millipore, Billerica, MA) containing 1mg/ml hyaluronidase (H4272, Sigma-Aldrich, St. Louis, MO), and cumulus-oocyte complexes were released into the medium. Purified TALEN mRNAs were mixed and diluted to a total concentration of 10ng/µl in microinjection buffer (10mM, Tris pH 7.4, 0.1mM EDTA). The diluted suspension was spun at high speed for five minutes, and a small aliquot was removed from the top for needle loading. An injection dish was prepared in a 60 mm petri dish (Falcon, Becton-Dickinson, NJ). Embryos were visualized on an inverted Nikon Eclipse Ti-U equipped with HMC optics, and embryo manipulation was accomplished with Leitz manual manipulators. Denuded zygotes were injected in one pronucleus with the mRNA suspension until the pronucleus visibly swelled. Embryos were then cultured overnight in KSOM (EMD Millipore) and transferred at the two-cell stage to recipient CD-1 females (#022, Charles River Laboratory). Embryo manipulation and pronuclear injection were carried out by the KUMC Gene Targeting Institutional Facility. All procedures conducted were approved by the KUMC Institutional Animal Care and Use Committee (IACUC) in accordance with the Guide for the Care and Use of Laboratory Animals.

Live pups arising from the gene-targeted embryos were assessed for deletion mutations in *Casp1* initially by PCR and sequencing of a 242 bp region within the *Casp1* gene, spanning from a site within exon 6 to a site within the following intron. Primers used for this were 5'-ATCCAGGAGGGAATATGTGG-3' and 5'- CAGGAATCAACCCCAAACAC-3'. One mutation having a 28-bp deletion that included the coding region for the active-site cysteine residue of Caspase-1 and the splice donor of exon 6 was bred to homozygosity in the RC/RC mouse. To confirm that mice were knocked-out for the expression of *Casp1*, kidney tissues of WT and the *Casp1* deletion mouse were used for RNA purification and cDNA synthesis, as described. PCR was then performed to generate a DNA fragment spanning *Casp1* exons 4-9 using primers 5'-ACCCTCAAGTTTTGCCCTTT-3' and 5'-TCAGCAGTGGGCATCTGTAG-3' and the products were analyzed on agarose gels and visualized following staining with ethidium bromide (0.5 μg/ml).

Hydroxychloroquine treatment of $Pkd1^{RC/RC}$ mice

RC/RC mice on the BALB/c background (12 females and 12 males) were treated with human-grade hydroxychloroquine sulfate (Intas Pharmaceuticals Limited, Gujarat. India) dissolved in the drinking water to provide 10 mg/kg/d, assuming a mouse weight of 20 g and a daily water consumption of 4 ml/d. Mice were treated from the time of weaning (P21-22) until 4 months of age.

Tissue harvest, analyses of fibrosis and cystic burden, and immunohistochemistry

Mice were weighed and euthanized by isofluorane inhalation and opening of the chest cavity, and blood was collected via heart puncture. The right kidney was quartered equally and frozen immediately in liquid N_2. The left kidney was placed into formalin, which was changed to 70% ethanol after 24 h. Sections (5 μm) from formalin-fixed, paraffin-embedded kidneys were stained with hematoxylin and eosin (Richard-Allan Scientific, Kalamazoo, MI) and/or with picrosirius red (no. 24901, Polyscience, Warrington, PA), and images were captured either on an Olympus BX41 microscope with the X2 objective using a Spot Idea camera and Spot Imaging Software (v5.2.5, Spot Imaging) or on an Aperio AT2 slide scanner (Leica Biosystems, Inc., Buffalo Grove, IL) at 40X resolution. The total number of cysts, cystic area and total area of the picrosirius red stained mid-sagittal kidney sections (one per mouse) were quantified from the Aperio AT2 images using Image J2 Fiji (NIH) after setting the threshold to distinguish between cysts and kidney tissue. Blood vessels were manually darkened to prevent them being counted as cysts. Cysts were defined as having a diameter of >50 μm. Cystic index was calculated as the ratio of total cystic area divided by the total area of the entire mid-sagittal kidney section. To measure fibrosis index, these same picrosirius-red stained sections captured on an Aperio AT2 slide scanner at 40X resolution were used. Fibrosis area was determined for the complete mid-sagittal kidney section after appropriate threshold settings to distinguish these areas from non-fibrotic tissue. Fibrosis index was calculated as the ratio of total fibrosis area divided by the total are of the mid-sagittal kidney section minus the cystic area.

To determine total kidney cystic volume, formalin-fixed kidneys were first soaked in the contrast agent Omnipaque™ (iohexol) (GE Healthcare Inc, Marlborough, MA NDC 0407-1414-89) diluted to 70 mg/ml in 70% ethanol. Kidneys were placed on a rocker for one week, to allow iohexol equilibration within the cystic spaces and the pelvis, and then subjected to high-resolution micro-computed tomography (Rowe, 2022, Saito and Murase, 2012). From these scans, contrast images of successive 6 μm sections of the entire kidney were obtained, which were then rendered into Z-stacks from which 3D volumetric data of the contrast-containing spaces (cysts plus pelvis) were then determined. Following raw data acquisition and computer reconstruction, images were

contoured and defined using Scanco software with image analysis thresholds described below to distinguish contrast-containing spaces from the surrounding kidney parenchyma. Total kidney volume and pelvic volume was quantified following the manual placement of guide outlines delineating the kidney or pelvis, respectively, in approximately 16 images of the Z-stack , and the software morph function was used to apply these outlines to all the acquired images. Cystic volume was determined by subtracting pelvic volume from the total contrast-containing volume for each kidney and was presented as a percentage of the total kidney volume.

The specifications for the renal cyst analysis "batch-control" file were as follows: (1) energy intensity of 45 kVp, 88 µA and 4 W; (2) FOV/Diameter of 12 mm; (3) voxel (VOX) resolution size of 6 µM; and (4) integration time of 300 mS. For 3D image and quantitative analysis, a script file were set to Gauss Sigma = 0.8 and Gauss Support = 1.0. Scanning thresholds for the renal structures ere: (1) for cysts, lower threshold = 350 permille, upper threshold 434 permille; and (2) for renal parenchyma, lower threshold = 240 permille, upper threshold = 350 permille. Acquired data was then exported to an Excel spreadsheet and statistics software packages (GraphPAD Prism, Statistica, SPSS) for final calculations.

For immunohistochemistry of Ki67, kidney sections (5 µm) from formalin-fixed, paraffin-embedded kidneys were processed and incubated with anti-Ki67 antibody (RM-9106, Thermo Scientific, Rochester, NY), as described previously (Swenson-Fields et al., 2013) and images were captured on an Aperio AT2 slide scanner (Leica Biosystems, Inc.,Buffalo Grove, IL). Using a 40X objective setting, the Ki-67+ cells, cyst cells and interstitial cells were counted in 4-5 non-overlapping hpf per kidney section and the % Ki67+/cyst cells and % Ki67+/interstitial cells was determined for each field.

Blood urea nitrogen measurement.

Each blood sample collected was stored on ice for at least 30 min, and serum was isolated by centrifugation at 18,000 x g for 8 min at 4°C followed by a second spin of the supernatant for 8 min. Blood urea nitrogen (BUN) was measured using the QuantiChrom Urea Assay Kit (BioAssay Systems, Hayward, CA).

Quantitative RT-PCR

Kidney tissue samples or cells (mouse and human) were lysed either by the addition of RLT lysis buffer from the RNeasy Miniprep kit (no. 74106, Qiagen, Valencia, CA) or the addition of TRIzol reagent (no. 15596026, ThermoFisher Scientific) and homogenized using a Pro200 homogenizer (Cole-Palmer, Vernon Hills, IL). Total RNA from these lysates was purified according to either the RNeasy Miniprep kit or TRIzol reagent directions. Final steps for all RNA preparations included DNase digestion (no. 79254, Qiagen, Valencia, CA) followed by column purification using RNeasy Miniprep columns. The quality of each RNA sample was determined with an Agilent Tape Station 4200 by the KUMC Genome Sequencing Facility, and RNAs with an RNA integrity number (RIN) of at least 6 were used for quantitative RT-PCR. cDNA was synthesized using the High-Capacity cDNA RT kit (no. 2021-0731, ThermoFisher Scientific) and quantitative RT-PCR analysis was performed on A Bio-Rad CFX96 real-time PCR cycler using IQ SYBR Green Supermix (no. 17088862, Bio-Rad Laboratories). The DART-PCR program (Peirson et al., 2003) was used to determine the efficiency of each primer pair and this information was then used to calculate the mRNA relative abundance of target genes according to methods described by (Pfaffl, 2001) and normalized to the mean of the reference gene used. Samples of genes with qRT-PCR Cqs greater than 35 were analyzed on agarose gels and visualized following staining with ethidium bromide (0.5 µg/ml) to provide semi-

quantitative PCR results. The sequences of primers used for qRT-PCR are listed in Supplementary Table 1.

RNA Seq

Total RNA was purified from kidney tissue samples collected from WT, RC/RC and RC/RC mice knocked-out for *Casp1* expression (RC/RC:*Casp1* KO). Kidney tissue samples of each mouse type, which were all C57BL/6J strains, were from 4 different mice (2 males and 2 females) that were 6 months of age. RNA samples were sent to Novagene for RNASeq and data analyses, which included identification of differentially expressed genes (DEGs), cluster analyses of DEGs, and KEGG (Kyoto Encyclopedia of Genes and Genomes) pathway enrichment analyses, among others. For the generation of the heatmap showing the normalized expression of individual samples, the raw counts for each sample were normalized using the R package DeSeq2 (Love et al., 2014).

Cells and tissues

Preparation of primary immune cells from mouse kidneys and spleens

Whole kidneys or spleens from female RC/RC or WT mice (6-9 month) were collected into ice-cold RPMI containing penicillin (200 U/ml), streptomycin (260 μg/ml) prior to mincing with a sterile razor blade and digestion for 30 min with collagenase type I (2 mg/ml in RPMI, no. LS004197, Worthington Biochemical, Lakewood, NJ) and DNase-1 (100U/ml, no. D5025, Sigma-Aldrich, Saint Louis, MO) at 37°C with shaking (150 rpm). Equal volume autoMacs Rinsing solution (no. 130-091-222, Miltenyi Biotec) containing 0.5% BSA (no. BP1605, Fisher Scientific; Pittsburgh, PA) (BSA/Macs) was added, and samples were strained sequentially through a 100 μM then a 40 μM strainer and centrifuged at (300 x g, 5 min). Pellets were washed twice and resuspend in BSA/Macs prior to immune cell enrichment by density separation using Lympholyte M (Cedarlane, Burlington, NC) followed by washing/suspension in BSA/Macs. The immune cells enriched from kidneys were then prepared for flow cytometry and/or isolation of specific cell types. Immune cells enriched from spleens were incubated overnight in RPMI-1640 media (R8758, Sigma-Aldrich, St. Louis, MO) containing 10% FBS, 200 μM L-glutamine and Pen/Strep, to which M-CSF and GM-CSF (5 mg/ml each, no. 416-ML and no. 415-ML, Bio-Techne Corporation, Minneapolis, MN) was added.

Primary cells obtained from the cavities of cysts on the surfaces of ADPKD kidneys (ADPKD cells) and those obtained from cortical tubule fragments from non-cystic kidneys (NHK cells), human pooled cyst fluids and renal tissues were supplied by the PKD Research Biomaterials and Cellular Models Core at KUMC, the ethical protocols of which comply with federal regulations and were approved by the KUMC Institutional Review Board. Cyst fluid was thawed and particulates removed by centrifugation at 1000 x g for 10 min. ADPKD and NHK cells were cultured up to 2 passages in DMEM/F-12 (Cellgro 15-090-CV, Mediatech, Manassas, VA) supplemented with 5% FBS, 15 mM HEPES, 5 μg/ml insulin, 5 μg/ml transferrin, and 5 ng/ml sodium selenite (ITS, CB40351; Fisher Scientific; Pittsburgh, PA) plus penicillin (100 U/ml), streptomycin (130 μg/ml) (Pen/Strep). THP-1 monocytes were maintained in RPMI-1640 media (R8758, Sigma-Aldrich, St. Louis, MO) containing 10% FBS, 200 μM L-glutamine and Pen/Strep.

Flow cytometry and isolation of primary renal immune cells

The purity of immune cells enriched from kidneys was assessed by FACS analysis of cells incubated with FITC-labeled anti-mouse CD45 antibodies (no. 103108, BioLegend, San Diego, CA) for 45 min after an initial 20 min incubation in an Fc-receptor blocking antibody (no.

553142, BD Biosciences, Haryana, India). CD45+ or CD11c+ cells were purified by magnetic bead separation according to manufacturer's instructions (no. 130-052-301 and no. 130-1080338, Miltenyi Biotec, Gladbach, Germany), the purity of which was assessed by FACS analysis using FITC-labeled anti-mouse CD45 (no. 103108, BioLegend, San Diego, CA) and PE-labeled anti-CD11c (no. 565909, BD Biosciences, Haryana, India) antibodies. To test for IL-1β production, purified CD45+ or CD11c+ cells (100,000 of each) or equal numbers of immune cells remaining following depletion by the respective type of magnetic bead separation were seeded in 96-well plates and cultured for 24 h. Conditioned media (CM) from these cells were collected and assayed by ELISA for IL-1β.

Inflammasome priming, activation and HCQ effects on cells in vitro

To test for an inflammasome priming response, ADPKD cells were treated with the bacterial products, either LPS (1.0 µg/ml, no. ALX-581-012-L011, Enzo Life Sciences, New York, NY) or Pam3CSK4 (100 ng/ml, no. 4633, Bio-Techne Corporation, Minneapolis, MN), for 24 h followed by lysis and RNA purification. To test for NLRP3 inflammasome activation, ADPKD cyst cells and THP-1 monocytes were primed with LPS (1.0 µg/ml) and/or PAM3CSK4 (100 ng/ml) for 24 h followed by the addition of nigericin (20µg/ml, no. N7143, MilliporeSigma, Saint Louis, MO) or media only for 2 h, at which time cells and CM were collected for analyses. To test HCQ-effects on inflammasome-induced production of cytokines in THP-1 monocytes, cells were primed with 100 ng/ml LPS for 3 h and then washed and resuspended in serum-free media. Hydroxychloroquine sulfate (no. 5092720001, MilliporeSigma, Saint Louis, MO) was added at various concentrations for 15 min followed by the addition of ATP (5 mM; GE27-2056-01, MilliporeSigma, Saint Louis, MO) for 45 min prior to the collection of CM for ELISAs of IL-1β and IL-18. Activation of the NLRP3 inflammasome in spleen immune cells to generate control immunoblot samples was carried out by priming with LPS (100 ng/ml, no. ALX-581-012-L011, Enzo Life Sciences, New York, NY) in serum-free media for 3 h followed by the addition of nigericin (10µg/ml, no. N7143, MilliporeSigma, Saint Louis, MO) for 45 min. CM was collected, concentrated using Amicron 10K centrifugal filter units (no. UFC501024, MilliporeSigma, Saint Louis, MO) and prepared for immunoblot analyses. To test for HCQ-effects on inflammasome production of IL-1β in spleen immune cells, cells were first washed and resuspended in serum-free media. They were then primed with LPS (1µg/ml) for 3 h, during which HCQ (various concentrations) was added (after 1 h). ATP (2mM) was then added for 2 h prior to collection of the CM, which used either for ELISA of IL-1β or was concentrated (500 µl/ sample) using Amicron 10K centrifugal filter units (no. UFC501024, MilliporeSigma, Saint Louis, MO) and prepared for immunoblot analyses.

Antibodies and ELISA kits

For immunoblots, antibodies specific for NLRP3 (no. 15101), human Caspase-1 (no. 3866) and YAP (no. 140747) were obtained from Cell Signaling, Cell Signaling Technology (Danvers, MA); immunoblot antibodies for tubulin (no. T5201, Sigma-Aldrich, St. Louis, MO), mouse Caspase-1 (no. AG-20B-0042, Adipogen Life Sciences, Liestal, Switzerland), mouse IL-1β (no. AF401; R&D Systems, Minneapolis, MN) Myc (no. 32072, Abcam, Cambridge, UK) and Gapdh (no. sc32233, Santa Cruz Biotechnology, Inc., Dallas, TX) were also used. All antibodies were diluted according to the manufacturers recommendations. Immunoblot bands were detected using the Odyssey system from Li-Cor Biotechnology (Lincoln, NE) following incubation with the appropriate secondary antibodies. Immunoblots of mouse cells and products reacted with anti-Caspase-1 antibody (no. AG-20B-0042, Adipogen Life Sciences, Liestal, Switzerland) were incubated with a biotin-conjugated secondary antibody (no. 115-065-071,

Jackson ImmunoResearch, Westgrove, PA) followed by a fluorescently-labeled streptavidin (no. 016-650-084, Jackson ImmunoResearch) prior to band detection. Immunoblots reacted with anti-IL-1β (no. AF401; R&D Systems) were incubated also with a biotin-conjugated secondary antibody (no. 305-065-045, Jackson ImmunoResearch) followed by a fluorescently-labeled streptavidin (no. 016-650-084, Jackson ImmunoResearch).

IL-1β and IL-18 in cyst fluids of human ADPKD patients, pre-cleared by low-speed centrifugation (1000 x g for 10 min) and vortexed for at least 10 sec to remove viscosities, were measured in duplicate using ELISA kits from eBioscience (no. 88-7261, San Diego, CA) and Medical and Biological Laboratories (no. 7620, Nagoya, Japan), respectively, according to the manufacturer's protocol. All other ELISA measurements were carried out in duplicate also. Human IL-1β in culture supernatants of ADPKD, NHK cells and THP-1 cells from experiments shown in Supplementary Figure 1 were measured using an ELISA kit from R&D Systems (no. DY201-05, Minneapolis, MN). Mouse IL-1β in culture supernatants from primary renal immune cells shown in Figure 3 was measured using an ELISA kit also from R&D Systems (no. DY401-05, Minneapolis, MN). In the experiments shown in Supplementary Figure 7, human IL-1β and IL-18 was measured in THP-1 culture supernatants using ELISA Cartridge #SPCKB-PS-000216 and # SPCKB-PS-000501 from Bio-techne (Minneapolis, MN), respectively, and mouse IL-1β was measured in spleen immune cell culture supernatants using ELISA cartridge #SPCKB-MP-003377 also from Bio-techne (Minneapolis, MN). The measurement of IL-18 in sera was conducted using ELISA kit from MilliporeSigma (RAB0810, Saint Louis, MO).

References

Arroyo, J., Escobar-Zarate, D., Wells, H. H., Constans, M. M., Thao, K., Smith, J. M., Sieben, C. J., Martell, M. R., Kline, T. L., Irazabal, M. V., Torres, V. E., Hopp, K. & Harris, P. C. 2021. The genetic background significantly impacts the severity of kidney cystic disease in the Pkd1(RC/RC) mouse model of autosomal dominant polycystic kidney disease. *Kidney Int,* 99**,** 1392-1407.

Atala, A., Freeman, M. R., Mandell, J. & Beier, D. R. 1993. Juvenile cystic kidneys (jck): a new mouse mutation which causes polycystic kidneys. *Kidney Int,* 43**,** 1081-5.

Cermak, T., Doyle, E. L., Christian, M., Wang, L., Zhang, Y., Schmidt, C., Baller, J. A., Somia, N. V., Bogdanove, A. J. & Voytas, D. F. 2011. Efficient design and assembly of custom TALEN and other TAL effector-based constructs for DNA targeting. *Nucleic Acids Res,* 39**,** e82.

Hopp, K., Ward, C. J., Hommerding, C. J., Nasr, S. H., Tuan, H. F., Gainullin, V. G., Rossetti, S., Torres, V. E. & Harris, P. C. 2012. Functional polycystin-1 dosage governs autosomal dominant polycystic kidney disease severity. *J Clin Invest,* 122**,** 4257-73.

Liu, S., Lu, W., Obara, T., Kuida, S., Lehoczky, J., Dewar, K., Drummond, I. A. & Beier, D. R. 2002. A defect in a novel Nek-family kinase causes cystic kidney disease in the mouse and in zebrafish. *Development,* 129**,** 5839-46.

Love, M. I., Huber, W. & Anders, S. 2014. Moderated estimation of fold change and dispersion for RNA-seq data with DESeq2. *Genome Biol,* 15**,** 550.

Peirson, S. N., Butler, J. N. & Foster, R. G. 2003. Experimental validation of novel and conventional approaches to quantitative real-time PCR data analysis. *Nucleic Acids Res,* 31**,** e73.

Pfaffl, M. W. 2001. A new mathematical model for relative quantification in real-time RT-PCR. *Nucleic Acids Res,* 29**,** e45.

Rowe, P. S. M., E. M.; Yu, A. L.; Stubbs, J. R. 2022. Correction of Vascular Calcification and Hyperphosphatemia in Chronic Kidney Disease Rats Treated with ASARM peptide. *Kidney 360,* Publish Ahead of Print.

Saito, S. & Murase, K. 2012. Ex vivo imaging of mouse brain using micro-CT with non-ionic iodinated contrast agent: a comparison with myelin staining. *Br J Radiol,* 85**,** e973-8.

Swenson-Fields, K. I., Vivian, C. J., Salah, S. M., Peda, J. D., Davis, B. M., Van Rooijen, N., Wallace, D. P. & Fields, T. A. 2013. Macrophages promote polycystic kidney disease progression. *Kidney Int,* 83**,** 855-64.
